# Miro, a Rho GTPase genetically interacts with Alzheimer's disease-associated genes (*Tau*, *Aβ_42_* and *Appl*) in *Drosophila melanogaster*

**DOI:** 10.1242/bio.049569

**Published:** 2020-09-03

**Authors:** Komal Panchal, Anand Krishna Tiwari

**Affiliations:** Genetics and Developmental Biology Laboratory, Department of Biological Sciences and Biotechnology, Institute of Advanced Research (IAR), Koba, Gandhinagar, Gujarat 382426, India

**Keywords:** Miro, Tau, Aβ_42_, Appl, Mitochondria, Alzheimer's disease

## Abstract

Miro (mitochondrial Rho GTPases), a mitochondrial outer membrane protein, facilitates mitochondrial axonal transport along the microtubules to facilitate neuronal function. It plays an important role in regulating mitochondrial dynamics (fusion and fission) and cellular energy generation. Thus, Miro might be associated with the key pathologies of several neurodegenerative diseases (NDs) including Alzheimer's disease (AD). In the present manuscript, we have demonstrated the possible genetic interaction between Miro and AD-related genes such as *Tau*, *Aβ_42_* and *Appl* in *Drosophila melanogaster*. Ectopic expression of *Tau*, *Aβ_42_* and *Appl* induced a rough eye phenotype, defects in phototaxis and climbing activity, and shortened lifespan in the flies. In our study, we have observed that overexpression of *Miro* improves the rough eye phenotype, behavioral activities (climbing and phototaxis) and ATP level in AD model flies. Further, the improvement examined in AD-related phenotypes was correlated with decreased oxidative stress, cell death and neurodegeneration in *Miro* overexpressing AD model flies. Thus, the obtained results suggested that *Miro* genetically interacts with AD-related genes in *Drosophila* and has the potential to be used as a therapeutic target for the design of therapeutic strategies for NDs.

This article has an associated First Person interview with the first author of the paper.

## Abstract (precis)

**Summary:** Our study suggests that overexpression of *Miro* improves the Alzheimer's disease-related pathologies by decreasing the rough eye phenotype, oxidative stress, apoptosis and neurodegeneration and increasing the lifespan, body weight, phototaxis and climbing activity in the *Drosophila* model of Alzheimer's disease.

## INTRODUCTION

*Mitochondrial Rho GTPase* (*Miro*) is an evolutionary conserved mitochondrial outer membrane protein that plays a pivotal role in mitochondrial axonal transport and maintenance of mitochondrial dynamics (fusion and fission) (Kay et al., 2018; Lee and Lu, 2014; Reis et al., 2009; Tang, 2016). Miro forms a major protein complex with Milton (adaptor protein) and, kinesin and dynein (motor proteins) to facilitate mitochondrial bi-directional axonal transport such as anterograde (cell body to axon) and retrograde (axon to cell body) transport (Cai and Sheng, 2009; Panchal and Tiwari, 2018). The involvement of *Miro* in the impairment of mitochondrial axonal transport that ultimately leads to neurodegeneration has previously been reported (López–Doménech et al., 2018; Russo et al., 2009; Tang, 2016).

Alzheimer's disease (AD) is the second most common neurodegenerative disorder characterized by the formation of extracellular Aβ_42_ plaques (amyloidogenic cleavage of APPL protein) by β and γ-secretase and intracellular neurofibrillary tangles composed of hyperphosphorylated tau protein (Binder et al., 2005; O'Brien and Wong, 2011). Various molecular changes have been reported in AD, which includes early metabolic changes, neuronal death, memory loss, cognitive decline, mitochondria dysfunction and defective mitochondrial axonal transport (Mosconi et al., 2014; Tan and Azzam, 2017). The fruit fly, *Drosophila melanogaster*, is commonly used as a model organism to explore the molecular details of several neurological diseases including AD. In the *Drosophila* model of AD, neuronal death results in rough eye phenotype, learning and memory loss, impaired climbing and phototaxis activity with reduced lifespan (Jahn et al., 2011; McGurk et al., 2015; Simon et al., 2012). Further, weight loss, an early metabolic change, associated with Aβ-mediated toxicity in hypothalamic neurons induces a reduction in body weight in AD model flies (Cova et al., 2016; Sergi et al., 2013). Moreover, several studies have suggested that overexpression of AD-related genes (*Appl*, *Aβ_42_* and *Tau*) in *Drosophila* induced caspase-dependent cell death (apoptosis) via increasing cellular stress and mitochondrial dysfunction resulting in reduced ATP level and enhanced oxidative stress (Cai et al., 2005; Pérez et al., 2018; Park et al., 2013). Thus, studying the different parameters such as behavior, cell death, mitochondrial function including ATP level and oxidative stress would be very useful to study the molecular details of AD-related pathologies.

A study by Iijima-Ando et al. (2009) showed that overexpression of *Aβ_42_* in AD model flies results in the reduction of mitochondria numbers in axons and dendrites, and increases mitochondria accumulation in somata of the neurons. This mitochondrial mislocalization exacerbated by *Miro* mutation ultimately enhances Aβ_42_-induced behavioral deficits in *Drosophila*. Moreover, knockdown of *Miro* in AD model flies has been reported to enhance the tau-induced neurodegeneration by increasing the tau phosphorylation in AD-related site S262 by PAR1 kinase (Iijima-Ando et al., 2012), suggesting that *Miro* might play an important role in the modulation of AD-related pathologies.

Interestingly, an axonal transport study in *Drosophila* has revealed that *Drosophila* Miro is functionally homologous to human Miro 1 and Miro 2 proteins (Tang, 2016; Kay et al., 2018). The *Drosophila* mitochondrial axonal transport protein complex Miro/Milton/kinesin is also homologous to mammalian Miro/TRAK/KIF5 protein complex (Lee and Lu, 2014; Tang, 2016). These similarities between *Drosophila* and human mitochondrial axonal transport proteins make *Drosophila* a powerful model organism to study the mitochondria dysfunction related pathologies in AD (Kay et al., 2018; Russo et al., 2009).

As mentioned above, Miro plays a key role in neurodegeneration by regulating the mitochondrial axonal transport, but the molecular details of how *Miro* interacts with AD-related genes (*Tau*, *Aβ_42_* and *Appl*) are not well understood yet. Thus, in the current study, we studied the possible genetic interaction between the mitochondrial axonal transport gene *Miro* and AD-related genes (*Tau*, *Aβ_42_* and *Appl*) in *Drosophila*.

## RESULTS

### Ectopic expression of AD-associated genes (*Tau*, *Aβ_42_* and *Appl*) showed AD-related pathologies in *Drosophila*

*Tau*, *Aβ_42_* and *Appl* (AD-related genes) are involved in the pathogenesis of AD and their ectopic expression results in several phenotypic/behavioral abnormalities such as rough eye phenotype, phototaxis and climbing defect, and reduced survival and body weight in *Drosophila* (Fernández-Moriano et al., 2015; Gistelinck et al., 2012; Iijima-Ando and Iijima, 2010; Peng et al., 2015; Roy and Jackson, 2014).

To examine the AD-related pathologies such as rough eye phenotype and behavioral changes (phototaxis and locomotor), AD model flies were used in the present study. We have expressed AD-related genes such as *UAS-Tau_WT,_ UAS- Aβ_42_(Human)* and *UAS-Appl^RNAi^* in the eyes of flies using pan-retinal *GMR-GAL4* [*GMR-GAL4-UAS-Tau_WT_/+_,_ GMR-GAL4-UAS-Aβ_42_(Human)/+* and *GMR-GAL4/+;UAS-Appl^RNAi^/+*] and expressed other AD genes such as *UAS-Aβ_42_E693G* and *UAS-APP.C99-UAS-MAPT* in the neurons using pan-neuronal *elav-Gal4^C155^* (*elav-Gal4^C155^/+;+/+;UAS-Aβ_42_E693G/*+ and *elav-Gal4^C155^/+;+/+;UAS-APP.C99-UAS-MAPT/+*).

**Fig. 1.**
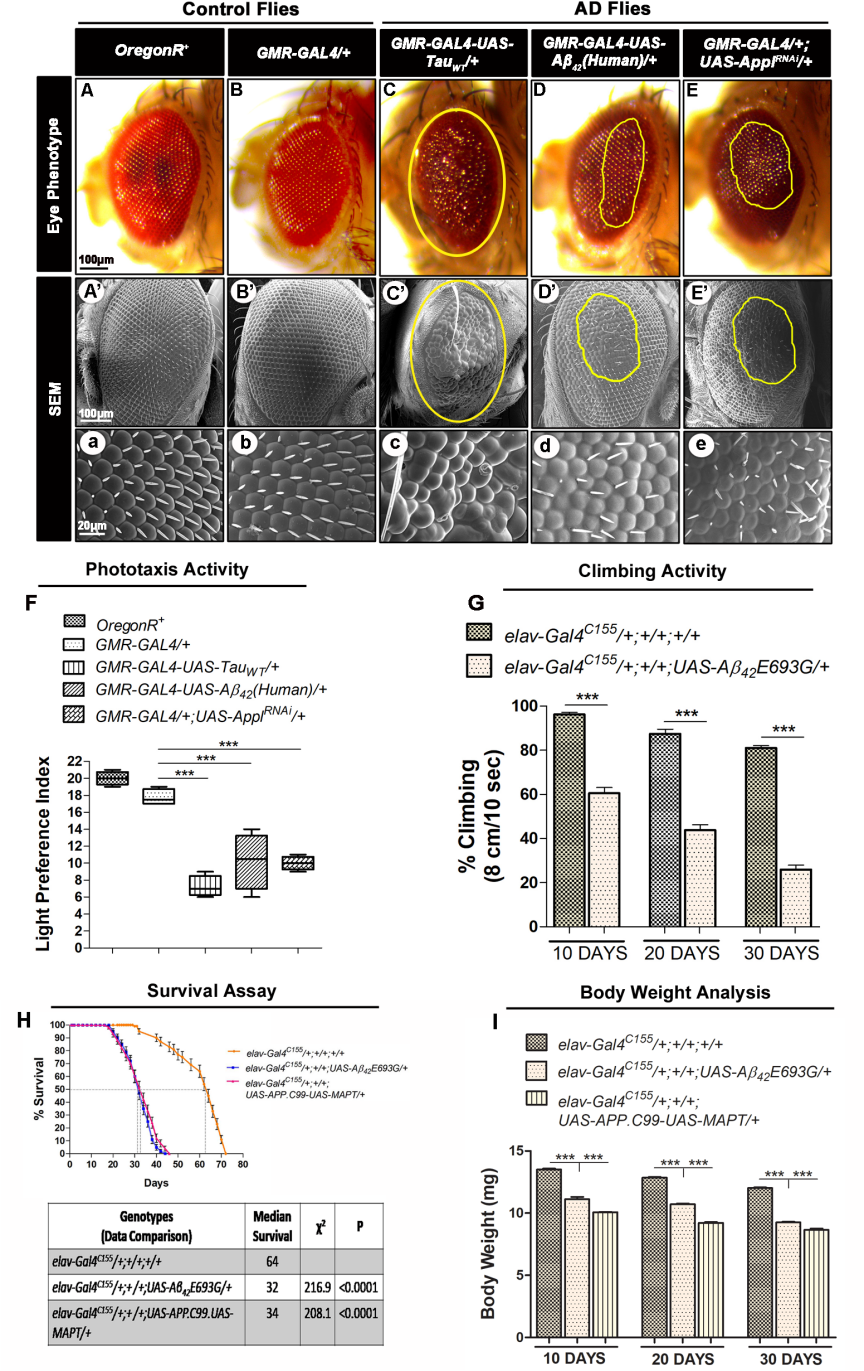
**AD related pathologies in *Drosophila*.** (A–E) Light microscopic and (A′–E′,a–e) SEM images of eyes of 10-day-old adult flies from *OregonR^+^* (wild-type control) (A,A′,a), *GMR- GAL4/+* (experimental control) (B,B′,b), *GMR-GAL4-UAS-TAU_WT_/+* (C,C′,c), *GMR-GAL4-UAS-Aβ_42_(Human)/+* (D,D′,d) and *GMR-GAL4/+; UAS-Appl^RNAi^/+* (E,E′,e). (a–e) are magnified images of SEM. Scale bar: 100 μm (A–E,A′–E′) and 20 μm (a–e). The yellow marked area shows degenerated part of eyes (C–E,C′–E′). *n*=50. (F) Phototaxis activity of 10-day-old control (*OregonR^+^*, *GMR-GAL4/+*) and AD model flies *GMR-GAL4-UAS-TAU_WT_/+*, *GMR-GAL4-UAS-Aβ_42_(Human)/+* and *GMR-GAL4/+; UAS-Appl^RNAi^/+*. Phototaxis activity presented as a light preference index. *n*=100. In the box and whisker plot, the box outlines show the upper and lower quartiles**.** (G) Histogram showing climbing activity [expressed as % climbing (8 cm 10 s^−1^)] of 10-, 20- and 30-day-old adult flies of *elav-Gal4^C155^/+;+/+;+/+* and *elav-Gal4^C155^/+;+/+;UAS-Aβ_42_E693G/*+. *n*=100. (H) Survival assay of *elav-Gal4^C155^/+;+/+;+/+* (yellow line), *elav-Gal4^C155^/+;+/+;UAS-Aβ_42_E693G/*+ (blue line) and *elav-Gal4^C155^/+;+/+;UAS-APP.C99-UAS-MAPT/+* (pink line)*. n*=100. The Kaplan–Meier survival test was performed and significance was determined by Montel-Cox log-rank test. A table indicating data comparison between control flies versus AD model flies with median lifespan (days), Chi-Square test (*χ^2^*) and *P*-value (Montel-Cox log-rank test). Data comparison: life span of AD model flies such as *elav-Gal4^C155^/+;UAS-Miro/+;UAS-Aβ_42_E693G/*+ (*P*<0.0001) and *elav-Gal4^C155^/+;UAS-Miro/+;UAS-APP.C99-UAS-MAPT/+* compared with control flies (*elav-Gal4^C155^/+;+/+;+/+*) (*P*<0.0001). (I) Body weight analysis of 10-, 20- and 30-day-old flies of *elav-Gal4^C155^/+;+/+;+/+, elav- Gal4^C155^/+;+/+;UAS-Aβ_42_E693G/*+ and *elav-Gal4^C155^/+;+/+;UAS-APP.C99-UAS-MAPT/+. n*=100. Error bars represent mean±s.e.m. Data significance was calculated by one-way ANOVA analysis with Tukey's test using GraphPad Prism 5.0 and is indicated as ***P<0.0001.

It was observed that the ectopic expression of AD-related genes in the fly eyes results in a degenerated eye phenotype in *Drosophila* (Fig. 1A–E,A′–E′,a–e). Further, the magnified view of *Drosophila* eye images from the AD model showed retinal degeneration along with disarrangements of ommatidia and bristles in the eye (Fig. 1c–e) as compared to wild type (*OregonR^+^*) and experimental control (*GMR-GAL4/+*) flies (Fig. 1a,b).

Phototaxis activity (expressed as light preference index) was also decreased in 10-day-old AD model flies [*GMR-GAL4-UAS-Tau_WT_/+_,_ GMR-GAL4-UAS-Aβ_42_(Human)/+* and *GMR-GAL4/+;UAS-Appl^RNAi^/+*] to 7.3, 10.3 and 10.0, respectively, as compared to *GMR-GAL4/+* flies which had a light preference index of 17.8 (Fig. 1F).

The climbing assay (to examine climbing deficits) was performed in 10-, 20- and 30-day-old AD model flies (*elav-Gal4^C155^/+;+/+;UAS-Aβ_42_E693G/*+). The climbing activity was significantly decreased to 60.56%, 43.76% and 25.92% in 10-, 20- and 30-day-old AD model flies, respectively, as compared to 10-, 20- and 30-day-old experimental control flies (*elav-Gal4^C155^/+;+/+;+/+*), which had climbing activity of 96.22%, 87.46% and 81.08%, respectively (Fig. 1G).

Furthermore, we performed the survival assays in AD model flies to check the lifespan (Fig. 1H). It was observed that the median lifespans of *elav-Gal4^C155^/+;+/+;UAS-Aβ_42_E693G/*+ and *elav-Gal4^C155^/+;+/+;UAS-APP.C99-UAS-MAPT/+* flies were significantly decreased to 32 and 34 days as compared to *elav-Gal4^C155^/+;+/+;+/+* flies, which had a median lifespan of 64 days (Fig. 1H).

The body weight analysis of 10-, 20- and 30-day-old AD model flies (*elav-Gal4^C155^/+;+/+;UAS-Aβ_42_E693G/*+ and *elav-Gal4^C155^/+;+/+;UAS-APP.C99-UAS-MAPT/+*) was also performed. It was observed that the body weights of 10-, 20- and 30-day-old (*elav-Gal4^C155^/+;+/+;UAS-Aβ_42_E693G/*+) flies were significantly decreased to 11.12 mg, 10.72 mg and 9.25 mg, respectively, as compared to similar age *elav-Gal4^C155^/+;+/+;+/+* flies, which had body weights of 13.5 mg, 12.85 mg and 12.02 mg, respectively (Fig. 1I). In the case of 10-, 20- and 30-day-old *elav-Gal4^C155^/+;+/+;UAS-APP.C99-UAS-MAPT/+* flies, body weight was significantly decreased to 10.07 mg, 9.22 mg, and 8.66 mg, respectively, as compared to similar age *elav-Gal4^C155^/+;+/+;+/+* flies, which had body weights of 13.5 mg, 12.85 mg and 12.02 mg, respectively (Fig. 1I).

Together, these results suggest that *Drosophila* models of AD used in the present study show AD-related pathologies (Fig. 1).

### Overexpression/knockdown of *Miro* alters the AD-related pathologies in *Drosophila*

As mentioned above, Miro plays a key role in mitochondrial axonal transport and dynamics (Guo et al., 2005; Saxton and Hollenbeck, 2012). The defect in mitochondrial axonal transport and dynamics are one of the key pathologies associated with AD. Thus, to find out the participation of Miro in AD, we have performed a genetic interaction study between the mitochondrial axonal transport gene *Miro* and the AD-associated genes (*Tau*, *Aβ_42_* and *Appl*) in *Drosophila*. The genetic interaction study was performed by crossing the AD model flies with *Miro* overexpressing/knockdown strains and examining the offspring for any phenotypic manifestation. Any alteration in the phenotype will suggest the possible genetic interaction between *Miro* and AD-associated genes in *Drosophila*. The genetic interaction study by enhancer and suppressor analysis is a key method for finding out the functional relationships between genes and pathways, and gives indispensable information regarding gene functions (Michaut and Bader, 2012; Thibault, 2011).

**Fig. 2.**
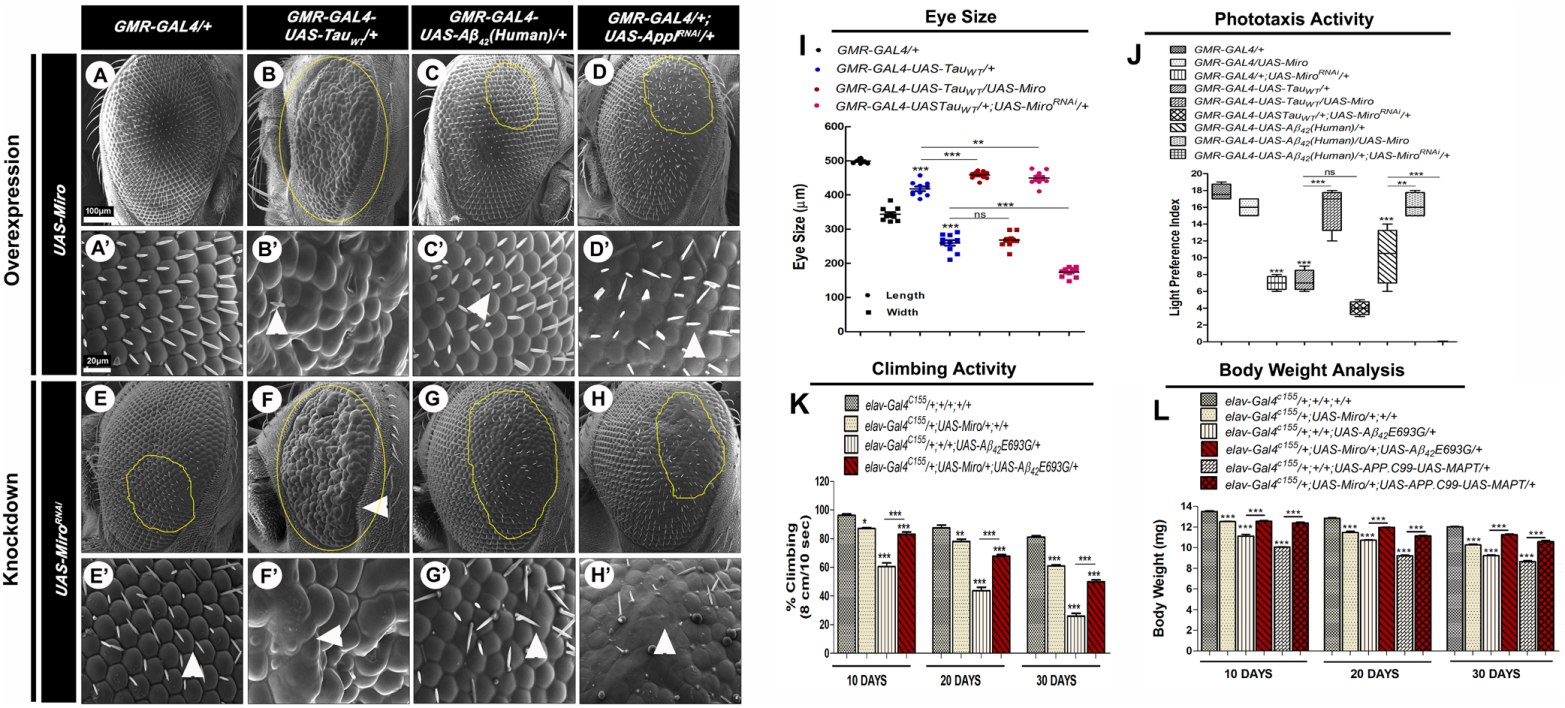
***Miro* modulates the AD-related pathologies in *Drosophila*.** (A–D) SEM images of eyes of 10-day-old *Miro* overexpressing adult flies from *GMR-GAL4/UAS-Miro* (A), *GMR-GAL4-UAS-TAU_WT_/UAS-Miro* (B), *GMR-GAL4-UAS-Aβ_42_(Human)/UAS-Miro* (C) and *GMR-GAL4/UAS-Miro; UAS-Appl^RNAi^/+* (D). Overexpression of *Miro* decreases the rough eye phenotype associated with AD model flies. (E–H) SEM images of eyes of 10-day-old *Miro* knockdown flies from *GMR-GAL4/+;UAS-Miro^RNAi^/+* (E), *GMR-GAL4-UAS-Tau_WT_/+;UAS-Miro^RNAi^/+* (F), *GMR-GAL4-UAS-Aβ_42_(Human)/+; UAS-Miro^RNAi^/+* (G) and *GMR-GAL4/+; UAS-Appl^RNAi^/UAS-Miro^RNAi^* (H). The knockdown of *Miro* enhances the rough eye phenotype associated with AD model flies. (A′–H′) are the magnified eye images of (A–H). Scale bar: 100 μm (A–H) and 20 μm (a–e). The yellow marked area showing the degenerated part of the eye. *n*=50. (I) Histogram showing the eye size (length and width) of *GMR-GAL4/+, GMR-GAL4-UAS-TAU_WT_/+, GMR-GAL4-UAS-TAU_WT_/UAS-Miro* and *GMR-GAL4-UAS-TAU_WT_/+; UAS-Miro^RNAi^/+* flies. *n*=10. (J) Box and whisker plot showing phototaxis activity (expressed as light preference index) of 10-day-old flies of *GMR-GAL4/+, GMR-GAL4/UAS-Miro, GMR-GAL4/+; UAS-Miro^RNAi^/+, GMR-GAL4-UAS-Tau_WT_/+, GMR-GAL4-UAS-Tau_WT_/UAS-Miro, GMR-GAL4-UAS-Tau_WT_/+;UAS-Miro^RNAi^/+, GMR-GAL4-UAS-Aβ_42_(Human)/+, GMR-GAL4-UAS-Aβ_42_(Human)/UAS-Miro* and *GMR-GAL4-UAS-Aβ_42_(Human)/+; UAS-Miro^RNAi^/+. n*=100. (K) Climbing activity (expressed as % climbing in 8 cm 10 s^−1^) of 10-, 20- and 30-day-old flies of *elav-Gal4^C155^/+;+/+;+/+, elav-Gal4^C155^/+;UAS-Miro/+;+/+, elav-Gal4^C155^/+;+/+;UAS-Aβ_42_E693G/*+ and *elav-Gal4^C155^/+;UAS-Miro/+;UAS-Aβ_42_E693G/*+. *n*=100. (L) Body weight analysis of 10-, 20- and 30-day-old flies of *elav-Gal4^C155^/+;+/+;+/+, elav-Gal4^C155^/+;UAS-Miro/+;+/+, elav-Gal4^C155^/+;+/+;UAS-Aβ_42_E693G/*+, *elav-Gal4^C155^/+;UAS-Miro/+;UAS-Aβ_42_E693G/+, elav-Gal4^C155^/+;+/+;UAS-APP.C99.UAS-MAPT/*+ and *elav-Gal4^C155^/+;UAS-Miro/+;UAS-APP.C99.UAS-MAPT/*+. *n*=100. Error bars represent mean±s.e.m. Data significance was calculated by one-way ANOVA analysis with Tukey's test using GraphPad Prism 5.0 and is indicated as: ns, non-significant; *P<0.05, **P<0.01, ***P<0.0001.

*Drosophila Miro* gene was overexpressed and knocked down in AD model flies genetic background using *UAS-Miro* and *UAS-Miro^RNAi^* fly lines, respectively. We did not observe any changes in the eye phenotype in flies overexpressing *Miro* alone (*GMR-GAL4/ UAS-Miro*) (Fig. 2A,A′) as compared to control *GMR-GAL4/+* flies (Fig. 1B,B′ and b) while there was a small extent of disarrangement of ommatidia and bristles observed in the *Miro* knockdown flies alone (*GMR-GAL4/+; UAS-Miro^RNAi^/+*) (Fig. 2E,E′).

It was discovered that the rough eye phenotype as well as ommatidial and bristles arrangements associated with AD model flies were significantly improved by *Miro* overexpression [*GMR-GAL4-UAS-Tau_WT_/UAS-Miro, GMR-GAL4-UAS-Aβ_42_(Human)/UAS-Miro* and *GMR-GAL4/UAS-Miro;UAS-Appl^RNAi^/+*] (Fig. 2B–D,B′–D′). Knockdown of *Miro* in AD model flies genetic background [*GMR-GAL4-UAS-Tau_WT_/+;UAS-Miro^RNAi^/+,GMR-GAL4-UAS Aβ_42_(Human)/+;UAS-Miro^RNAi^/+, GMR-GAL4/+;UAS-Appl^RNAi^/UAS Miro^RNAi^*] showed enhanced rough eye phenotype as well as ommatidial and bristles disarrangements (Fig. 2F–H,F′–H′).

The fly eye size analysis showed that the eye length and width of AD model flies (*GMR-GAL4-UAS-Tau_WT_/+*) was significantly decreased to 426.98 µm and 269.59 µm, respectively, as compared to *GMR-GAL4/+* flies, which had an eye length of 496.35 µm and eye width of 338.54 µm (Fig. 2I). The knockdown of *Miro* in the AD model flies genetic background (*GMR-GAL4-UAS-Tau_WT_/+;UAS-Miro^RNAi^/+*) significantly decreased the fly eye width to 182.12 µm as compared to the AD model flies having an eye width of 69.59 µm (Fig. 2I). This result indicated that knockdown of *Miro* enhanced the eye degeneration associated with AD model flies.

As mentioned above, the learning and memory defect directly affects the behavioral activities in the AD model flies (Chakraborty et al., 2011; Moloney et al., 2010; Nichols et al., 2012). Thus, we examined the effect of *Miro* overexpression/knockdown on the phototaxis activity of 10-day-old AD model flies by performing the phototaxis assay (Fig. 2J).

As shown in Fig. 2J, flies overexpressing *Miro* alone (*GMR-GAL4/UAS-Miro*) did not show any changes in light preference index while knockdown of *Miro* (*GMR-GAL4/+;UAS-Miro^RNAi^/+*) showed a significantly decreased light preference index of 7.0 as compared to *GMR-GAL4*/+ flies, which had a light preference index of 17.8 (Fig. 2J).

We observed that light preference index was significantly decreased to 7.25 and 10.25 in AD model flies [*GMR*-*GAL4-UAS-Tau_WT_/+* and *GMR-GAL4-UAS-Aβ_42_(Human)/+*, respectively] as compared to *GMR-GAL4/+* flies, which had a light preference index of 17 (Fig. 2J).

The light preference index was (7.25) of *GMR*-*GAL4-UAS-Tau_WT_/+* flies was restored to 16.0 by *Miro* overexpression (*GMR-GAL4-UAS-Tau_WT_/UAS-Miro*) while knockdown of *Miro* (*GMR-GAL4-UAS-Tau_WT_/+;UAS-Miro^RNAi^/+*) decreased the light preference index to 4.0 (Fig. 2J).

In the case of *GMR-GAL4-UAS-Aβ_42_(Human)/+* flies, they had a light preference index of 10.25, which was restored to 12.75 by *Miro* overexpression [*GMR-GAL4-UAS-Aβ_42_(Human)/UAS-Miro*]. Knockdown of *Miro* in AD flies genetic backgrounds (*GMR-GAL4-UAS-Aβ_42_(Human)/+;UAS-Miro^RNAi^*/+) decreased the light preference index to 0.03 (Fig. 2J).

Further, we checked the effect of *Miro* on the climbing activity associated with AD model flies. As shown in Fig. 2K, the climbing activity of 10-, 20- and 30-day-old *Miro* overexpressing flies (*elav-Gal4^C155^/+;UAS-Miro/+;+/*+) was significantly decreased to 87%, 78% and 61%, respectively, as compared to similar age *elav-Gal4^C155^/+;+/+;+/+* flies, which had climbing activity of 96.22%, 87.46% and 81.08%, respectively (Fig. 2K).

The climbing activity of 10-, 20- and 30-day-old AD model flies (*elav-Gal4^C155^/+;+/+;UAS-Aβ_42_E693G/*+) was significantly decreased to 60.5%, 43.76% and 25.92%, respectively, as compared to same aged *elav-Gal4^C155^/+;+/+;+/+* flies (Fig. 2K). While overexpression of *Miro* in AD model flies (*elav-Gal4^C155^/+;UAS-Miro/+;UAS-Aβ_42_E693G/*+) significantly increased the climbing activity to 83.18%, 67.84% and 49.84%, respectively, as compared to same aged *elav-Gal4^C155^/+;+/+;UAS-Aβ_42_E693G/*+ flies (Fig. 2K).

We also examined the effect of *Miro* overexpression on the body weight of AD model flies. As shown in Fig. 2J, the bodyweight of 10-, 20- and 30-day-old flies overexpressing *Miro* alone (*elav-Gal4^C155^/+; UAS-Miro/+;+/+*) was significantly decreased to 12.5, 11.47 mg and 10.27 mg, respectively, as compared to control *elav-Gal4^C155^/+;+/+;+/+* flies, which had body weight of 13.5 mg, 12.9 mg and 12.02 mg, respectively (Fig. 2L).

The body weight of 10-, 20- and 30-day-old (*elav-Gal4^C155^/+;+/+;UAS-Aβ_42_E693G/+*) flies was significantly decreased to 11.12 mg, 10.72 mg and 9.25 mg, respectively, which was increased to 12.56 mg, 11.98 mg and 11.26 mg, respectively, in a *Miro* overexpressing genetic background (*elav-Gal4^C155^/+;UAS-Miro/+;UAS-Aβ_42_E693G/+*) (Fig. 2L).

In the case of 10-, 20- and 30-day-old *elav-Gal4^C155^/+; +/+;UAS-APP.C99-UAS-MAPT/+* AD model flies, the body weight was significantly decreased to 10.07 mg, 9.22 mg and 8.66 mg, respectively, as compared to same aged *elav-Gal4^C155^/+;+/+;+/+* flies. While overexpression of *Miro* in an AD model fly genetic background (*elav-Gal4^C155^/+; UAS-Miro/+;UAS-APP.C99-UAS-MAPT/+*) increased the body weight to 12.39 mg, 11.15 mg and 10.59 mg, respectively (Fig. 2L).

### Overexpression of *Miro* increases the lifespan of AD model flies

**Fig. 3.**
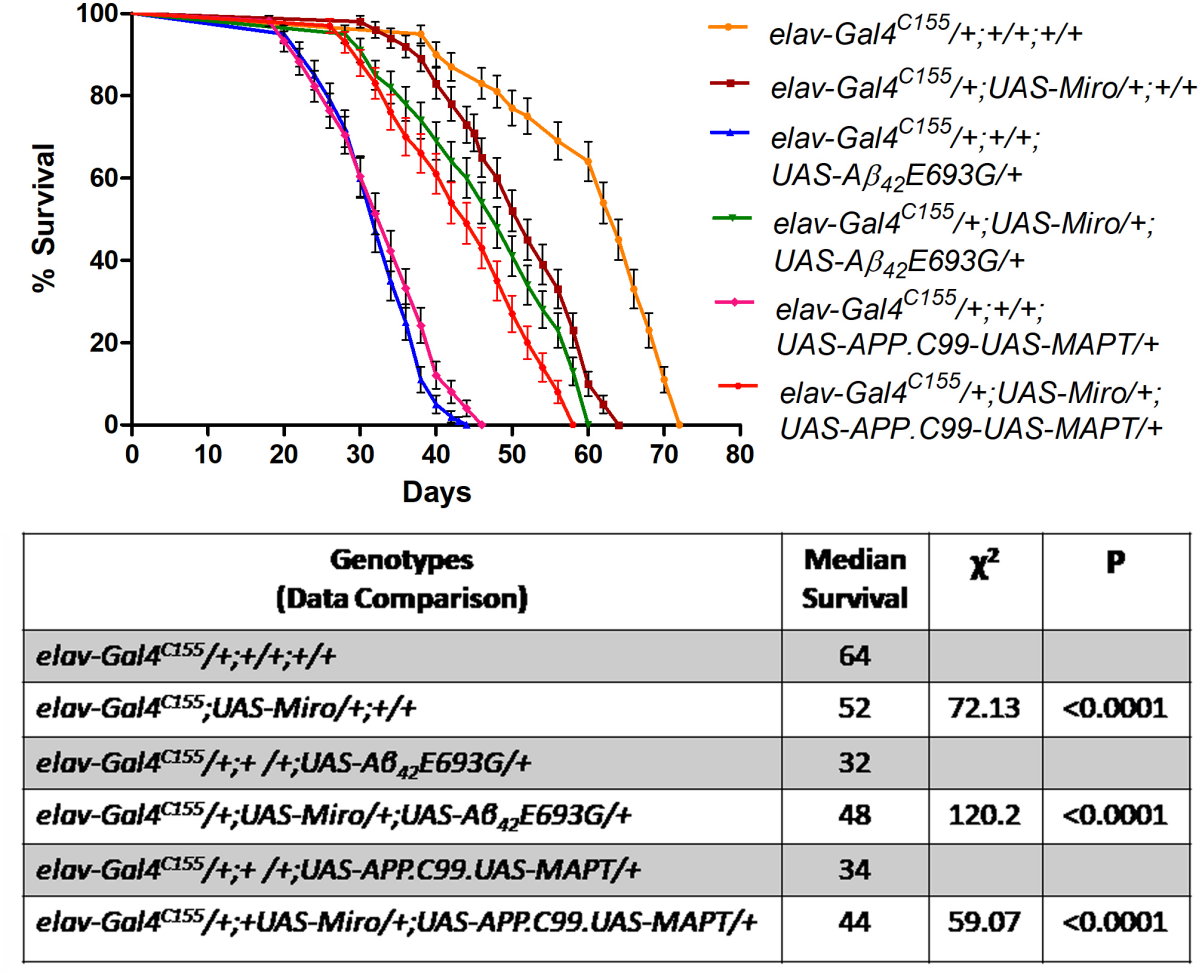
**Lifespan analysis of *Miro* overexpressing AD model flies.** Survival curve of *elav-Gal4^C155^/+;+/+;+/+* (yellow line), *elav-Gal4^C155^/+;UAS-Miro/+;+/+* (maroon line), *elav-Gal4^C155^/+;+/+;UAS-Aβ_42_E693G/+* (blue line), *elav-Gal4^C155^/+;+/+;UAS-APP.C99-UAS-MAPT/+* (pink line), *elav-Gal4^C155^/+;UAS-Miro/+; UAS-Aβ_42_E693G/+* (green line) and *elav-Gal4^C155^/+;UAS-Miro/+;UAS-APP.C99-UAS-MAPT/+* (orange line). *n*=100. The Kaplan–Meier survival test was performed and significance was determined by the Montel-Cox log-rank test using GraphPad Prism 5.0 software. A table indicating data comparison between all genotypes with median lifespan (days), Chi-Square test (χ^2^) and *P*-value (Montel-Cox log-rank test). Data comparison: lifespan of *elav-Gal4^C155^/+;UAS-Miro/+;+/+* compared with *elav-Gal4^C155^/+;+/+;+/+* (*P*<0.0001) and lifespan of Miro overexpressing AD model flies *elav-Gal4^C155^/+;UAS-Miro/+;UASAβ_42_E693G/+* (*P*<0.0001) and *elav-Gal4^C155^/+;UAS-Miro/+;UAS-APP.C99-UAS-MAPT/+* (*P*<0.0001) compared with AD model flies such as *elav-Gal4^C155^/+; UAS-Miro/+;UAS-Aβ_42_E693G/+* and *elav-Gal4^C155^/+;UAS-Miro/+;UAS-APP.C99-UAS-MAPT/+*, respectively.

As we have shown in Fig. 1H, AD model flies have a shortened lifespan as compared to control flies. Thus, we checked the effect of *Miro* overexpression on the median lifespan of AD model flies. We observed that median lifespan of *Miro* overexpressing flies (*elav-Gal4^C155^/+;UAS-Miro/+;/+/*) was significantly decreased as compared to *elav-Gal4^C155^/+;+/+;+/+* flies, which had a median lifespan of 64 days (Fig. 3). Further, we observed that the median lifespans of *Miro* overexpressing AD model flies (*elav-Gal4^C155^/+;UAS-Miro/+;UAS-Aβ_42_E693G/*+ and *elav-Gal4^C155^/+;UAS-Miro/+;UAS-APP.C99-UAS-MAPT/+*) were significantly extended to 48 days and 44 days, respectively, as compared to AD model flies (*elav-Gal4^C155^/+;UAS-Miro/+;UAS-Aβ_42_E693G/*+ and *elav-Gal4^C155^/+;UAS-Miro/+;UAS-APP.C99-UAS-MAPT/+*), which had median lifespans of 32 days and 34 days, respectively (Fig. 3).

### Overexpression of *Miro* modulates the cell death in eye imaginal discs of AD model flies

**Fig. 4.**
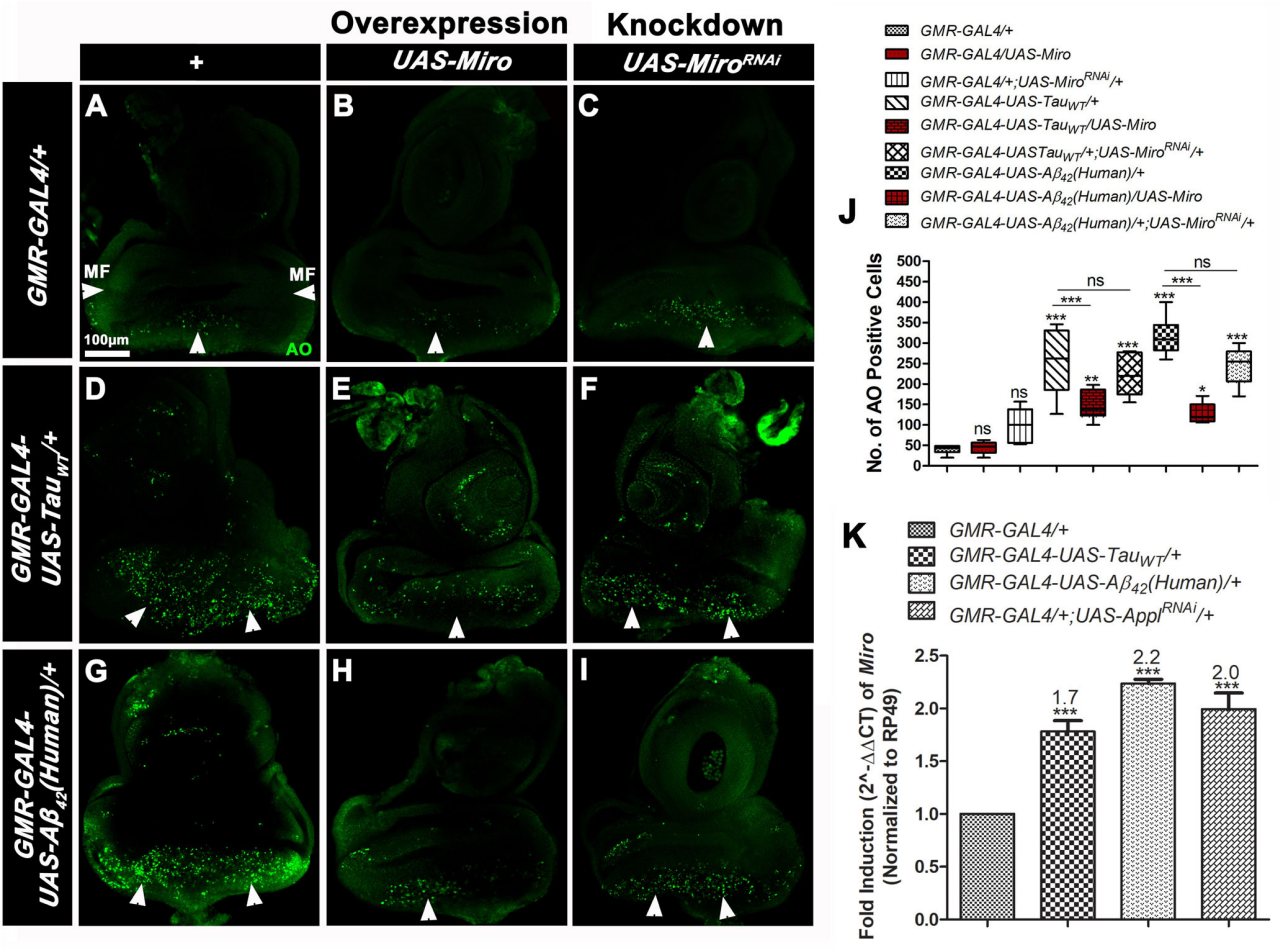
**AO staining in third instar larval eye discs of *Miro* overexpressing/knockdown AD model flies and quantitative real time PCR analysis of the *Miro* gene.** (A–I) Confocal images of AO stained third instar larval eye discs of *GMR-GAL4/+* (A), *GMR-GAL4/UAS-Miro* (B), *GMR-GAL4/+; UAS-Miro^RNA^^i^* (C), *GMR-GAL4-UAS-TAU_WT_/+* (D) *GMR-GAL4-UAS-TAU_WT_/UAS-Miro* (E), *GMR-GAL4-UAS-Tau_WT_/+;UAS-Miro^RNAi^/+* (F), *GMR-GAL4-UAS- Aβ_42_(Human)/+* (G), *GMR-GAL4-UAS-Aβ_42_(Human)/UAS-Miro* (H), *GMR-GAL4-UAS-Aβ_42_(Human)/+; UAS-Miro^RNAi^/+* (I). AO positive cells (dead cells) [posterior to the morphogenetic furrow (MF)] were indicated by white arrowheads. *n*=20. Scale bars: 100 µm (A–I). (J) Box and whisker plot showing average AO positive cells in third instar larval eye imaginal discs of each genotype. (K) The histogram showing quantitative real time PCR of *Miro* gene in 30-day-old adult flies heads of *GMR-GAL4/+, GMR-GAL4-UAS-Tau_WT_/+_,_ GMR-GAL4-UAS-Aβ_42_(Human)/+* and *GMR-GAL4/+;UAS-Appl^RNAi^/+* flies*. RP49* was used as an endogenous control. The quantification of AO positive cells was done by using ImageJ software, NIH, USA. Error bar represents mean±s.e.m. Data significance was calculated by one-way ANOVA analysis with Tukey's test using GraphPad Prism 5.0 and is indicated as: ns, non-significant; *P<0.05, **P<0.01, ***P<0.0001.

As shown in the above Fig. 2A–H, overexpression of *Miro* improved while knockdown of *Miro* potentiated the rough eye phenotype associated with AD model flies. Thus, to find out whether the rough eye phenotype was associated with ectopic cell death in eyes, we performed Acridine Orange (AO) staining in third instar larval eye imaginal discs (Fig. 4A–I) of experimental control (*GMR-GAL4/+*), *Miro* overexpressing (*GMR-GAL4/UAS-Miro*) and knockdown (*GMR-GAL4/+;UAS-Miro^RNAi/^+*) flies, AD model flies [*GMR-GAL4-UAS-Tau_WT_/+* and *GMR-GAL4-UAS-Aβ_42_(Human)/+*] and AD model flies with *Miro* overexpression [*GMR-GAL4-UAS-Tau_WT_/UAS-Miro* and *GMR-GAL4-UAS-Aβ_42_(Human)/UAS-Miro*] and *Miro* knockdown [*GMR-GAL4-UAS-Tau_WT_/+;UAS-Miro^RNAi^/+* and *GMR-GAL4-UAS-Aβ_42_(Human)/+;UAS-Miro^RNAi^/+*].

We observed that AD model flies showed excessive cell death (AO positive cells) posterior to the morphogenetic furrow (MF) in larval eyes (Fig. 4D,G,J) as compared to the *GMR-GAL4/+* flies showing few apoptotic cells (Fig. 4A,J). Overexpression of *Miro* in AD model flies (Fig. 4E,H,J) showed a significant reduction in cell death, while knockdown of *Miro* in AD model flies genetic background (Fig. 4F,I,J) did not show any significant changes in apoptotic cells as compared to the respective AD model flies. As shown in Fig. 4B and C, where *Miro* alone was overexpressed or knocked down did not show any changes in apoptotic cells as compared to *GMR-GAL4/+* flies. This result clearly suggests that overexpression of *Miro* modulates apoptosis in AD model flies.

As seen in the above results, overexpression of *Miro* modulates AD-related pathologies. Thus, we have checked *Miro* gene expression level in 30-day-old AD model flies by performing quantitative real-time PCR analysis. We observed that relative expression of the *Miro* gene was significantly increased in AD model flies [*GMR-GAL4-UAS-Tau_WT_/+_,_ GMR-GAL4-UAS-Aβ_42_(Human)/+* and *GMR-GAL4/+;UAS-Appl^RNAi^/+*] to 1.7-, 2.2- and twofold, respectively, as compared to *GMR-GAL4*/+ flies (Fig. 4K). This result indicated that the expression of AD genes significantly upregulates the *Miro* gene in *Drosophila*. It suggests that the function of Miro in AD-related pathologies is conserved in *Drosophila*.

### Overexpression of *Miro* decreases mitochondrial and cellular oxidative stress in AD model flies

**Fig. 5.**
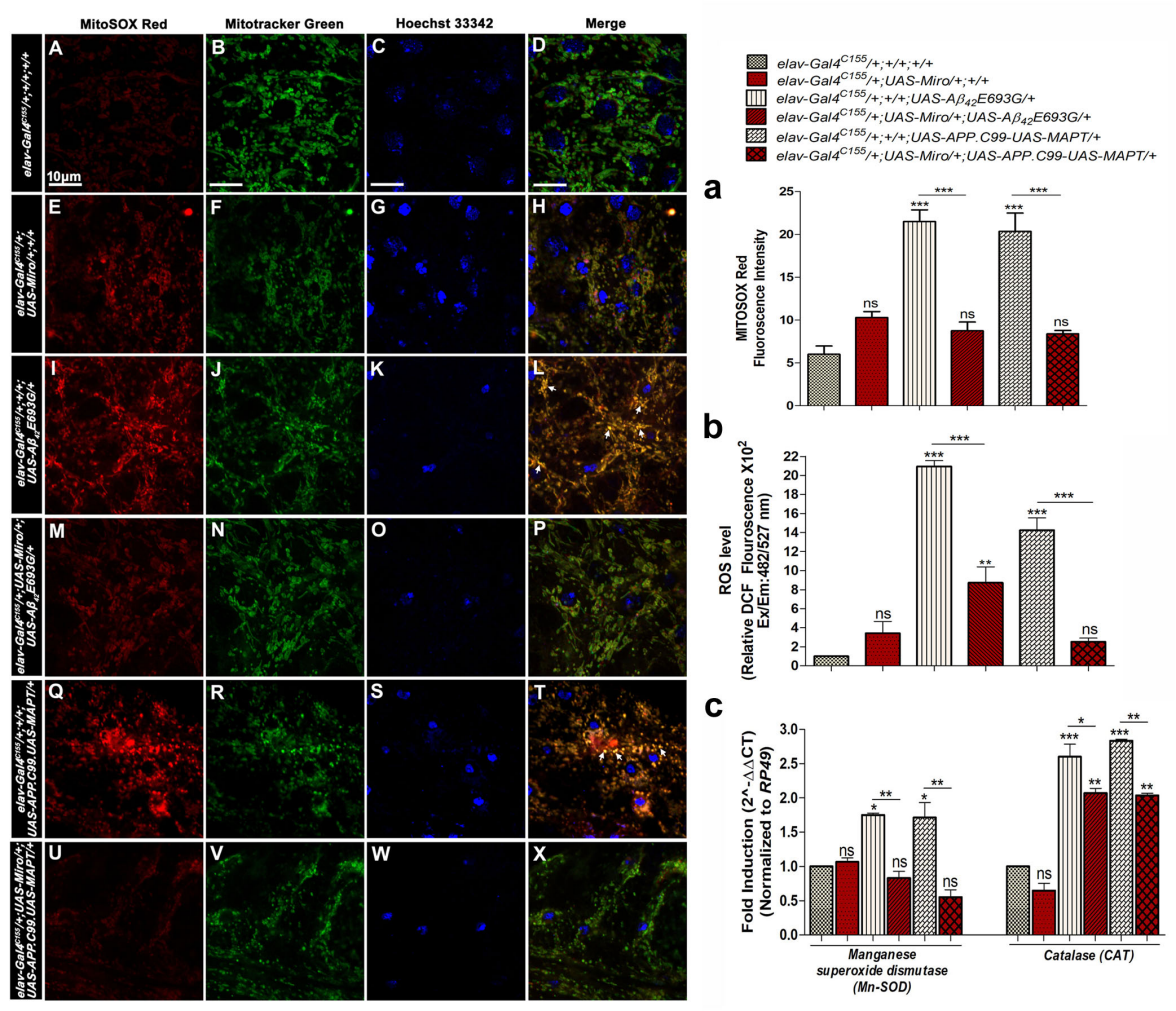
**Mitochondrial and cellular ROS level in *Miro* overexpressing AD model flies.** Confocal images of third instar larval brains showing MitoSOX Red staining (A,E,I,M,Q,U), MitoTracker Green (B,F,J,N,R,V), nuclear staining with Hoechst 33342 (C,G,K,O,S,W) in *elav-Gal4^C155^/+;+/+;+/+*, *elav-Gal4^C155^/+;UAS-Miro/+;+/+*, *elav-Gal4^C155^/+;+/+;UAS-Aβ_42_E693G/+*, *elav-Gal4^C155^/+;UAS-Miro/+;UAS-Aβ_42_E693G/*+, *elav-Gal4^C155^/+;+/+;UAS-APP.C99-UAS-MAPT/+* and *elav-Gal4^C155^/+;UAS-Miro/+;UAS-APP.C99-UAS-MAPT/+*, respectively. *n*=20 D, H, L, P, T, X are the merge images of A–C, E–G, I–K, M–O, Q–S and U–W, respectively. Colocalization in the merged images is shown by arrows. *n*=20. Scale bars: 10 µm (A–X). (a) Histogram showing average MitoSOX Red fluorescence intensity in third instar larval brains of each genotype. (b) Histogram showing cellular ROS level in 30-day-old adult flies heads of *elav-Gal4^C155^/+;+/+;+/+, elav-Gal4^C155^/+;UAS-Miro/+;+/+; elav-Gal4^C155^/+;+/+;UAS-Aβ_42_E693G/*+,*elav-Gal4^C155^/+;UAS-Miro/+;UAS-Aβ_42_E693G/*+, *elav-Gal4^C155^/+;+/+;UAS-APP.C99-UAS-MAPT/+* and *elav-Gal4^C155^/+;UAS-Miro/+;UAS-APP.C99-UAS-MAPT/+* flies by using DCF dye at Ex./Em. 482/527 nm and normalized to the amount of protein (µg). (c) Histogram showing the relative expression of *Mn-SOD* and *CAT* genes determined by quantitative real-time PCR in 30-day-old adult flies heads of*elav-Gal4^C155^/+;+/+;+/+, elav-Gal4^C155^/+;UAS-Miro/+;+/+; elav-Gal4^C155^/+;+/+;UAS-Aβ_42_E693G/*+,*elav-Gal4^C155^/+;UAS-Miro/+;UAS-Aβ_42_E693G/*+, *elav-Gal4^C155^/+;+/+;UAS-APP.C99-UAS-MAPT/+* and *elav-Gal4^C155^/+;UAS-Miro/+;UAS-APP.C99-UAS-MAPT/+* flies*.* MitoSOX Red fluorescence intensity was measured by using ImageJ software (NIH, USA). Error bar represents mean±s.e.m. Data significance was calculated by one-way ANOVA analysis with Tukey's test using GraphPad Prism 5.0 and is indicated as ns: non-significant, *P<0.05, **P<0.01, ***P<0.0001.

It is well known that oxidative stress and altered mitochondrial dynamics play a key role in AD pathogenesis (Wang et al., 2009; Zhu et al., 2013). Thus, to examine the status of mitochondria-mediated oxidative stress and the effect of overexpression/knockdown of *Miro* in AD model flies, we have performed MitoSOX Red staining to measure the mitochondrial ROS, MitoTracker Green to labelled mitochondria and Hoechst 33342 staining to visualize the nucleus in third instar larval brain of control and experimental group flies (Fig. 5A–X).

AD model flies (*elav-Gal4^C155^/+;+/+;UAS-Aβ_42_E693G/*+ and *elav-Gal4^C155^/+;+/+;UAS-APP.C99-UAS-MAPT/+*) showed a significant induction of MitoSOX Red fluorescence and increased co-localization (yellow) with MitoTracker Green staining suggesting a higher level of mitochondrial ROS production in both the AD model flies (Fig. 5I–L,Q–T,a), as compared to *elav-Gal4^C155^/+;+/+;+/+* flies (Fig. 5A–D,a). Overexpression of *Miro* in AD model flies such as *elav-Gal4^C155^/+;UAS-Miro/+;UAS-Aβ_42_E693G/*+ (Fig. 5M–P,a) and *elav-Gal4^C155^/+;UAS-Miro/+;UAS-APP.C99-UAS-MAPT/+* (Fig. 5U–X,a) showed a significant reduction of MitoSOX Red fluorescence suggesting that *Miro* overexpression helps in the reduction of mitochondrial ROS level in AD model flies.

We have also checked the cellular (cytosolic) ROS level in control and AD model flies alone and in the *Miro* overexpressing AD model flies genetic background using 2′,7′-dichlorodihydrofluorescein diacetate (DCFH-DA) dye. In the presence of cellular ROS, non-fluorescent H2DCFDA was oxidized and converted into highly fluorescent 2′,7′-dichlorofluorescein (DCF) (Kalyanaraman et al., 2012; Tetz et al., 2013). As shown in Fig. 5b, AD model flies (*elav-Gal4^C155^/+;+/+;UAS-Aβ_42_E693G/*+ and *elav-Gal4^C155^/+;+/+;UAS-APP.C99-UAS-MAPT/+*) showed strong fluorescence of DCF, suggesting higher cellular ROS production (Fig. 5b). While overexpression of *Miro* in AD model flies (*elav-Gal4^C155^/+;UAS-Miro/+;UAS-Aβ_42_E693G/*+ and *elav-Gal4^C155^/+;UAS-Miro/+;UAS-APP.C99-UAS-MAPT/+*) significantly decreased the DCF fluorescence in adult brain as compared to the control (Fig. 5b). The DCF fluorescence was not affected by overexpression of *Miro* alone (*elav-Gal4^C155^/+;UAS-Miro/+;+/+*) as compared to *elav-Gal4^C155^/+;+/+;+/+* flies (Fig. 5b).

To further validate the above observations, we have examined the effect of *Miro* overexpression on the anti-oxidant enzymes genes expression levels such as *Manganese Superoxide dismutase* (*Mn-SOD*) and *Catalase* (*CAT*) in 30-day-old AD model flies by performing quantitative real-time PCR analysis (Fig. 5c). It is known that the cooperative function of SOD and CAT helps in the protection against oxidative stress (Ighodaro and Akinloye, 2018; Luangwattananun et al., 2016). Mitochondrial Mn-SOD is also known as *Drosophila* SOD2, which clears mitochondrial ROS via eliminating the superoxide radical (Candas and Li, 2014). Mn-SOD cleaved superoxide radical and produce H_2_O_2_, which is further degraded into H_2_O and O_2_ by CAT enzyme (Candas and Li, 2014; Wang et al., 2018). It provides protection against cell death and plays a vital role in the protection from neurodegenerative diseases (Flynn and Melov, 2013; Niedzielska et al., 2016). Thus, this study evaluated the effect of *Miro* overexpression on *Mn-SOD* and *CAT* gene expression levels in AD model flies (Fig. 5c).

We observed the relative expression of mitochondrial *Mn-SOD* gene was significantly increased in AD model flies (*elav-Gal4^C155^/+;+/+;UAS-Aβ_42_E693G/*+ and *elav-Gal4^C155^/+;+/+;UAS-APP.C99-UAS-MAPT/+*) to 1.8- and 1.7-fold, respectively, as compared to *elav-Gal4^C155^/+;+/+;+/+* flies (Fig. 5c). This higher level of *Mn-SOD* gene expression was significantly decreased to 0.8- and 0.6-fold in *Miro* overexpressing AD model flies such as elav*-Gal4^C155^/+;UAS-Miro/+;UAS-Aβ_42_E693G/*+ and *elav-Gal4^C155^/+;UAS-Miro/+;UAS-APP.C99-UAS-MAPT/+*, respectively (Fig. 5c).

In the case of the *CAT* gene, the relative expression level of *CAT* in AD model flies (*elav-Gal4^C155^/+;+/+;UAS-Aβ_42_E693G/*+ and *elav-Gal4^C155^/+;+/+;UAS-APP.C99-UAS-MAPT/+*) was significantly increased to 2.6- and 2.8-fold, respectively, as compared to *elav-Gal4^C155^/+;+/+;+/+* flies (Fig. 5c). While overexpression of *Miro* in AD model flies (elav*-Gal4^C155^/+;UAS-Miro/+;UAS-Aβ_42_E693G/*+ and *elav-Gal4^C155^/+;UAS-Miro/+;UAS-APP.C99-UAS-MAPT/+*) significantly decreased the relative *CAT* gene expression level to 2.1- and twofold, respectively, as compared to the respective AD model flies (Fig. 5c).

As shown in Fig. 5c, overexpression of *Miro* alone did not affect the expression of any of the antioxidant enzymes.

The above results confirmed that overexpression of *Miro* decreases expression of the antioxidant Mn-SOD and CAT enzymes via reducing mitochondrial and cellular ROS in AD model flies. *CAT* expression level in *Miro* expressing AD model flies was still higher than in control flies. This suggests that the overexpression of *Miro* may affect mitochondrial ROS more than cellular ROS.

### Overexpression of *Miro* altered mitochondrial dynamics in AD model flies

**Fig. 6.**
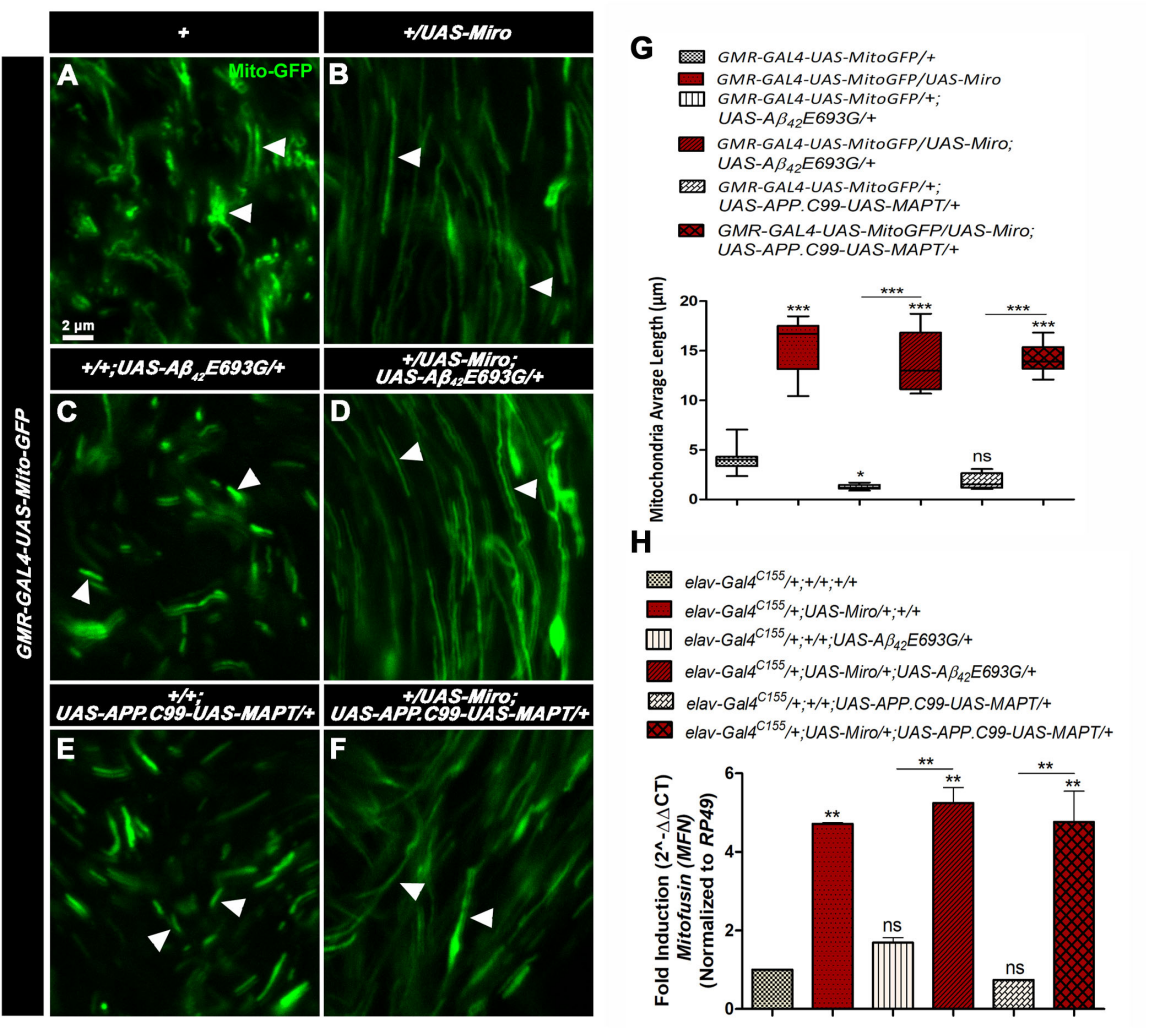
**Mitochondrial average length measurement.** (A–F) Confocal microscopy images showing mitochondrial length (indicated by white arrows) in third instar larval eye discs of *GMR-GAL4-MitoGFP/+* (A), *GMR-GAL4-MitoGFP/UAS-Miro* (B), *GMR-GAL4-UAS-Mito-GFP/+; UAS-Aβ_42_E693G/+* (C), *GMR-GAL4-UAS-Mito-GFP/UAS-Miro; UAS-Aβ_42_E693G/+* (D), *GMR-GAL4-UAS-Mito-GFP/+;UAS-APP.C99-UAS-MAPT/+* (E) and *GMR-GAL4-UAS-Mito-GFP/UAS-Miro;UAS-APP.C99-UAS-MAPT/+* (F). Scale bar: 2 µm (A–F), *n*=20. (G) Box whisker plot shows mitochondrial average length in third instar larval eye discs of each genotype. (H) Histogram showing the relative expression of *Mitofusin (Mfn)* gene determined by quantitative real-time PCR in 30-day-old adult flies heads of *elav-Gal4^C155^/+;+/+;+/+, elav-Gal4^C155^/+;UAS-Miro/+;+/+; elav-Gal4^C155^/+;+/+;UAS-Aβ_42_E693G/*+,*elav-Gal4^C155^/+;UAS-Miro/+;UAS-Aβ_42_E693G/*+, *elav-Gal4^C155^/+;+/+;UAS-APP.C99-UAS-MAPT/+* and *elav-Gal4^C155^/+;UAS-Miro/+;UAS-APP.C99-UAS-MAPT/+* flies. *RP49* was used as an endogenous control. Error bar represents mean±s.e.m. Data significance was calculated by one-way ANOVA analysis with Tukey's test using GraphPad Prism 5.0 software and is indicated as ns: non-significant, *P<0.05, **P<0.01, ***P<0.0001.

It has been shown that overexpression of *Miro* increased average length of mitochondria by increasing mitochondrial fusion (Kay et al., 2018; Tang, 2016). Thus, to examine the effect of Miro on mitochondrial dynamics in AD model flies, we have checked the average length of mitochondria using *GMR-GAL4-UAS-Mito-GFP* flies (Fig. 6A–G). As shown in Fig. 6, the average length of mitochondria was significantly increased in flies overexpressing *Miro* alone (*GMR-GAL4-UAS-Mito-GFP/UAS-Miro*) to 15.65 µm (Fig. 6B,G) as compared to control *GMR-GAL4-UAS-Mito-GFP/+* flies, which had an average mitochondrial length of 4.1 μm (Fig. 6A,G). Furthermore, the average length of mitochondria was decreased to 1.3 µm and 1.8 µm in AD model flies (*GMR-GAL4-UAS-Mito-GFP/+; UAS-Aβ_42_E693G/*+ and *GMR-GAL4-UAS-Mito-GFP/+;UAS-APP.C99-UAS-MAPT/+*), respectively, as compared to control flies (Fig. 6C,E and G). The average length of mitochondria was significantly increased to 13.8 µm and 14.3 µm in *Miro* overexpressing AD model flies (*GMR-GAL4-UAS-Mito-GFP/UAS-Miro; UAS-Aβ_42_E693G/*+ and *GMR-GAL4-UAS-Mito-GFP/ UAS-Miro;UAS-APP.C99-UAS-MAPT/+*), respectively (Fig. 6D,F,G).

Further, to examine whether increased mitochondrial average length is associated with mitochondrial fusion, we checked the expression level of the mitochondrial fusion gene, *Mitofusin* (*Mfn*) by performing quantitative real-time PCR analysis. As shown in Fig. 6H the relative expression level of the *Mfn* gene was increased to 4.7-fold in flies overexpressing *Miro* alone (*elav-Gal4C155/+;UAS-Miro/+;+/+*) as compared to *elav-Gal4^C155^/+;+/+;+/+* flies (Fig. 6H). The relative expression level of the *Mfn* gene in AD model flies (*elav-Gal4^C155^/+;+/+;UAS-Aβ_42_E693G/*+ and *elav-Gal4^C155^/+;+/+;UAS-APP.C99-UAS-MAPT/+*) was significantly decreased to 1.7- and 0.7-fold, respectively (Fig. 6H), while overexpression of *Miro* in AD model flies (*elav-Gal4^C155^/+;UAS-Miro/+;UAS-Aβ_42_E693G/*+ and *elav-Gal4^C155^/+;UAS-Miro/+;UAS-APP.C99-UAS-MAPT/+*) showed significantly increased mitochondrial average length to 5.2- and 4.8-fold, respectively (Fig. 6H). This result suggests that overexpression of *Miro* modulates mitochondrial dynamics via increasing the average length of mitochondria and altering the *Mfn* gene expression level in AD model flies.

### Overexpression of *Miro* increases the ATP level in AD model flies

**Fig. 7.**
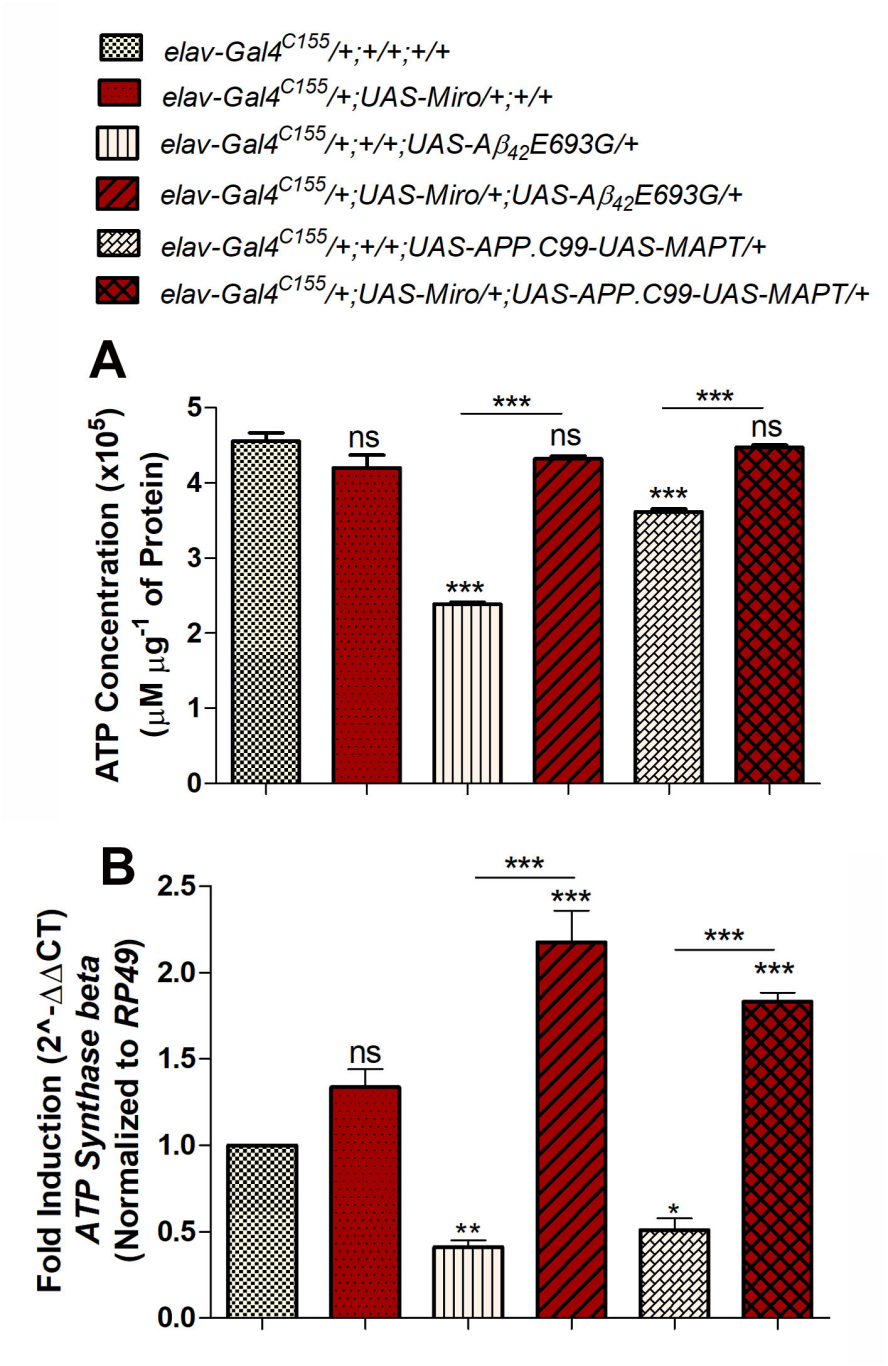
**ATP quantification of *Miro* overexpressing AD model flies.** (A) Histogram showing ATP concentration (µM µg^−1^ of protein) of 30-day-old adult flies heads of *elav-Gal4^C155^/+;+/+;+/+, elav-Gal4^C155^/+;UAS-Miro/+;+/+, elav-Gal4^C155^/+;+/+;UAS-Aβ_42_E693G/*+, *elav-Gal4^C155^/+;UAS-Miro/+;UAS-Aβ_42_E693G/*+, *elav Gal4^C155^/+;+/+;UAS-APP.C99-UAS-MAPT/+* and *elav-Gal4^C155^/+;UAS-Miro/+;UAS-APP.C99-UAS-MAPT/+*. (B) Histogram showing the relative expression of the *ATP Synthase beta* gene determined by quantitative real-time PCR in 30-day-old adult flies heads of*elav-Gal4^C155^/+;+/+;+/+, elav-Gal4^C155^/+;UAS-Miro/+;+/+; elav-Gal4^C155^/+;+/+;UAS-Aβ_42_E693G/*+,*elav-Gal4^C155^/+;UAS-Miro/+;UAS-Aβ_42_E693G/*+, *elav-Gal4^C155^/+;+/+;UAS-APP.C99-UAS-MAPT/+* and elav*-Gal4^C155^/+;UAS-Miro/+;UAS-APP.C99-UAS-MAPT/+* flies. *RP49* used as an endogenous control. Error bar represents mean±s.e.m. Data significance was calculated by one-way ANOVA analysis with Tukey's test using GraphPad Prism 5.0 software and is indicated as: ns, non-significant; *P<0.05, **P<0.01, ***P<0.0001.

As discussed above, overexpression of *Miro* increased mitochondrial fusion via increasing mitochondrial average length and the *Mitofusin* gene expression in AD model flies (Fig. 6). It has been reported that the fusion of mitochondria might result in an increased level of ATP production (Rambold et al., 2011; Song and Hwang, 2019). Thus, we examined the effect of *Miro* on ATP level in 30-day-old AD model flies (Fig. 7A). As shown in Fig. 7A, the ATP level in flies overexpressing *Miro* alone (elav*-Gal4^C155^/+;UAS-Miro/+;+/+*) was 4.2×10^5^ µM µg^−1^ of protein, which was similar to the control flies (*elav-Gal4^C155^/+;/+;+/+*) that had 4.6×10^5^ µM µg^−1^ of protein (Fig. 7A). In the case of AD model flies (*elav-Gal4^C155^/+;+/+;UAS-Aβ_42_E693G/*+ and *elav-Gal4^C155^/+;+/+;UAS-APP.C99-UAS-MAPT/+*), the ATP was significantly decreased to 2.4×10^5^ and 3.6×10^5^ µM µg^−1^ of protein as compared to control flies (Fig. 7A). This decreased ATP in AD model flies was significantly increased to 4.3×10^5^ and 4.5×10^5^ µM µg^−1^ of protein in *Miro* overexpressing AD model flies such as elav*-Gal4^C155^/+;UAS-Miro/+;UAS-Aβ_42_E693G/*+ and *elav-Gal4^C155^/+;UAS-Miro/+;UAS-APP.C99-UAS-MAPT/+*, respectively (Fig. 7A).

To further confirm this, we have checked the expression level of the *ATP synthase beta* gene by performing quantitative real-time PCR in 30-day-old adult fly heads of control and experimental group flies. As shown in Fig. 7B, we did not find any change in relative expression of *ATP synthase beta* in the flies overexpressing *Miro* alone (*elav-Gal4^C155^/+;UAS-Miro/+;+/+*) as compared to *elav-Gal4^C155^/+;+/+;+/+* control flies (Fig. 7B). Further, we observed the relative expression of *ATP synthase beta* was decreased to 0.4- and 0.5-fold in AD model flies such as *elav-Gal4^C155^/+;+/+;UAS-Aβ_42_E693G/*+ and *elav-Gal4^C155^/+;+/+;UAS-APP.C99-UAS-MAPT/+*, respectively, as compared to the control flies (Fig. 7B). The decreased relative expression of the *ATP synthase* gene was increased to 2.2- and 1.8-fold in *Miro* overexpressing AD model flies such as *elav-Gal4^C155^/+;UAS-Miro/+;UAS-Aβ_42_E693G/*+ and *elav-Gal4^C155^/+;UAS-Miro/+;UAS-APP.C99-UAS-MAPT/+*, respectively (Fig. 7B).

Together, these results suggest that overexpression of *Miro* increases the energy level in the form of ATP in AD model flies.

### Overexpression of *Miro* decreases cell death and neurodegeneration in the brain of AD model flies

**Fig. 8.**
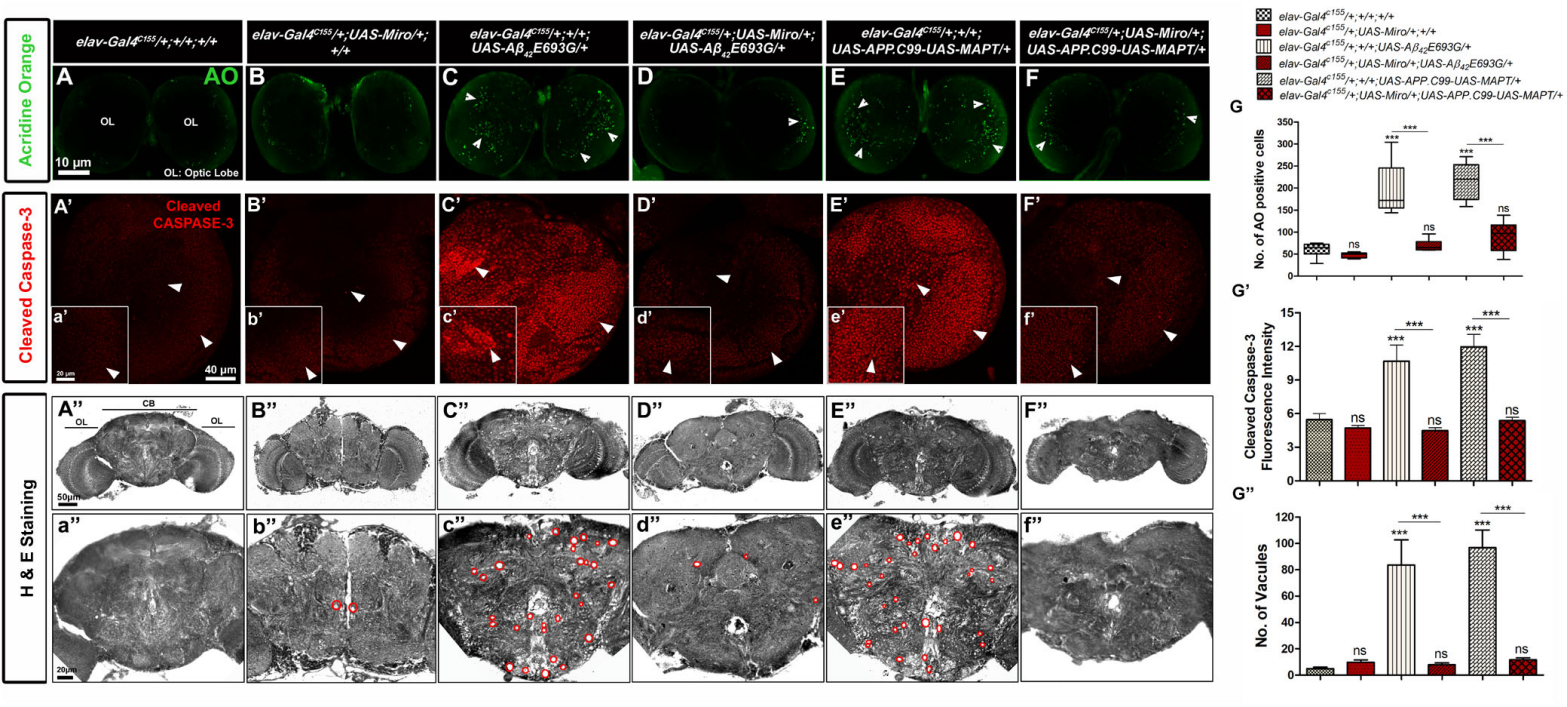
**Cell death and neurodegeneration analysis of *Miro* overexpressing AD model flies.** (A–F) Confocal images showing AO staining in third instar larval brains of *elav-Gal4^C155^/+;+/+;+/+* (A), *elav-Gal4^C155^/+;UAS-Mio/+;+/+* (B), *elav-Gal4^C155^/+;+/+;UAS-Aβ_42_E693G/*+ (C), *elav-Gal4^C155^/+;UAS-Miro/+;UAS-Aβ_42_E693G/*+ (D), *elav-Gal4^C155^/+;+/+;UAS-APP.C99-UAS-MAPT/+* (E), *elav-Gal4^C155^/+;UAS-Miro/+;UAS-APP.C99-UAS-MAPT/+* (F) flies. White arrowheads indicate AO positive cells in optic lobes (OL) of third instar larval brains. Scale bar: 10 µm (A–F), *n*=20. (G) Box and whisker plot showing the number of AO positive cells in third instar larval brains of each genotype. (A′–F′) Confocal images showing anti-cleaved-caspase-3 staining in third instar larval brain of *elav-Gal4^C155^/+;+/+;+/+* (A′), *elav-Gal4^C155^/+;UAS-Mio/+;+/+* (B′), *elav-Gal4^C155^/+;+/+;UAS-Aβ_42_E693G/*+ (C′), *elav-Gal4^C155^/+;UAS-Miro/+;UAS-Aβ_42_E693G/*+ (D′), *elav-Gal4^C155^/+;+/+;UAS-APP.C99-UAS-MAPT/+* (E′), *elav-Gal4^C155^/+;UAS-Miro/+;UAS-APP.C99-UAS-MAPT/+* (F′) flies. Scale bar: 40 µm (A′–F′). (a′–f′) Magnified confocal images of (A′–F′). Scale bar: 20 µm (a′–f′). *n*=20. White arrowheads indicate caspase positive cells (A′–F′, a′–f′). (G′) Histogram showing average fluorescence intensity of cleaved caspase-3 in third instar larval brains of each genotype. Cleaved caspase-3 fluorescence intensity was measured by using ImageJ software, NIH, USA. (A″–F″) Gray scale images of H&E stained paraffin sections of 30-day-old flies brains of *elav-Gal4^C155^/+;+/+;+/+* (A″), *elav-Gal4^C155^/+;UAS-Mio/+;+/+* (B″), *elav-Gal4^C155^/+;+/+;UAS-Aβ_42_E693G/*+ (C″), *elav-Gal4^C155^/+;UAS-Miro/+;UAS-Aβ_42_E693G/*+ (D″), *elav-Gal4^C155^/+;+/+;UAS-APP.C99-UAS-MAPT/+* (E″), *elav-Gal4^C155^/+;UAS-Miro/+;UAS-APP.C99-UAS-MAPT/+* (F″). Scale bar: 50 µm (A″–F″). (a″–f″) Magnified images of central region of the adult fly brains (A″–F″). Red-colored round shape indicates vacuoles (neurodegeneration) (a″–f″). Scale bar: 20 µm (a″–f″), *n*=10. (G″) The histogram shows an average number of vacuoles in each genotype of adult brains. Quantification of AO positive cells and the number of vacuoles was done by using ImageJ software (NIH, USA). Error bar represents mean±s.e.m. Data significance was calculated by one-way ANOVA analysis with Tukey's test using GraphPad Prism 5.0 and is indicated as: ns, non-significant, and ***P<0.0001.

We further examined the effect of *Miro* overexpression on cell death in AD model flies by staining third instar larval brain with AO, an apoptosis marker (Fig. 8A–F). As shown in Fig. 8B, we did not observe any changes in apoptosis in flies overexpressing *Miro* alone (*elav-Gal4^C155^/+;UAS-Miro/+;+/+*) (Fig. 8B,G), while cell death was significantly increased in *elav-Gal4^C155^/+;+/+;UAS-Aβ_42_E693G/*+ and *elav-Gal4^C155^/+;+/+;UAS-APP.C99-UAS-MAPT/+* flies (Fig. 8C,E,G) as compared to *elav-Gal4^C155^/+;+/+;+/+* control flies (Fig. 8A,G). Overexpression of *Miro* in AD model flies (*elav-Gal4^C155^/+;UAS-Miro/+;UAS-Aβ_42_E693G/*+ and *elav-Gal4^C155^/+;UAS-Miro/+;UAS-APP.C99-UAS-MAPT/+*) significantly decreased cell death (Fig. 8D,F,G) in the larval brain.

To further confirm the above result, we performed anti-cleaved caspase-3 staining in third instar larval brains of *Miro* overexpressing AD model flies. Caspase-3 is a typical cell death marker and an important mediator of programmed cell death (apoptosis) (Kumar and Tiwari, 2018; Porter and Jänicke, 1999). As shown in Fig. 8B′,b’ and G′ cleaved caspase-3 fluorescence intensity in flies overexpressing *Miro* alone (*elav-Gal4^C155^/+;UAS-Miro/+;+/+*) was similar to the *elav-Gal4^C155^/+;+/+;+/+* flies (Fig. 8A′,a′,G′). We observed a significant increase in cleaved caspase-3 fluorescence intensity in AD model flies (*elav-Gal4^C155^/+;+/+;UAS-Aβ_42_E693G/*+ and *elav-Gal4^C155^/+;+/+;UAS-APP.C99-UAS-MAPT/+*) (Fig. 8C′,c′,E′,e′,G′) as compared to *elav-Gal4^C155^/+;+/+;+/+* flies (Fig. 8A′,a′,G′). The overexpression of *Miro* in *elav-Gal4^C155^/+;UAS-Miro/+;UAS-Aβ_42_E693G/*+ and *elav-Gal4^C155^/+;UAS-Miro/+;UAS-APP.C99-UAS-MAPT/+* flies significantly decreased the cleaved caspase-3 fluorescence intensity (Fig. 8D′,d′,F′,f′,G′). This observation supports our above result that overexpression of *Miro* is significantly involved in the reduction in apoptosis (caspase-dependent) in AD model flies. These results suggest that *Miro* is notably involved in modulating the AD-related apoptosis in *Drosophila*.

Further, to check the neurodegeneration status in the *Miro* overexpressing AD model flies, we performed Hematoxylin and Eosin (H&E) staining in the histological sections of *Miro* overexpressing AD model flies and counted the number of vacuoles in the adult brain of control and experimental group flies. We observed that the number of vacuoles (neurodegeneration) was significantly increased in *elav-Gal4^C155^/+;+/+;UAS-Aβ_42_E693G/*+ and *elav-Gal4^C155^/+;+/+;UAS-APP.C99-UAS-MAPT/+* to 83.7 and 96.9, respectively (Fig. 8C″,c″,E″,e″,G″), as compared to *elav-Gal4^C155^/+;/+;+/+* flies, which had 4.9 number of vacuoles (Fig. 8A″,a″,G″). The increased number of vacuoles in AD model flies was significantly decreased to 7.8 and 11.6 by *Miro* overexpression such as elav*-Gal4^C155^/+;UAS-Miro/+;UAS-Aβ_42_E693G/*+ (Fig. 8D″,d″,G″) and *elav-Gal4^C155^/+;UAS-Miro/+;UAS-APP.C99-UAS-MAPT/+*, respectively (Fig. 8F″,f″,G″).

## DISCUSSION

Mitochondrial dynamics and mitochondrial axonal transport play a crucial role in neuronal growth and survival (Lovas and Wang, 2013; Mandal and Drerup, 2019). Several studies have suggested that altered mitochondrial dynamics and improper axonal transport are the early events of the onset of many neurodegenerative diseases (Guo et al., 2020; Kay et al., 2018). Miro is the sole protein that plays a vital role in the bi-directional mitochondrial axonal transport such as anterograde transport (from cell body to axon) and retrograde transport (from axon to cell body) via forming a major protein complex with Milton (adaptor protein), kinesin and dynein (motor proteins) (Russo et al., 2009; Cai and Sheng, 2009; Panchal and Tiwari, 2018). Miro provides ATP in the axons via facilitating the mitochondrial anterograde transport and promotes neuronal survival, while retrograde transport of mitochondria helps in the elimination of the damaged mitochondria via mitophagy (Russo et al., 2009; Guo et al., 2005). The various roles of Miro in synapses are to fulfill the ATP demand, maintaining the Ca^2+^ buffering and bioenergetics facilitated by mitochondrial axonal transport, which helps in the proper neurotransmission and neuronal survival (Lee and Lu, 2014). Thus, any alteration in Miro function may be associated with neurodegenerative disease conditions including AD (Wang et al., 2011; Lee and Lu, 2014). It has been demonstrated that knockdown of *Miro* induced mislocalization of mitochondria in the neurons that results in the accumulation of mitochondria in the cell body of neurons (Iijima-Ando et al., 2009). Apart from this, Miro also maintains mitochondria structure via regulating mitochondrial dynamics (fusion and fission) (Lee and Lu, 2014). Thus, it was inferred that Miro might play an important role in the modulation of AD-related pathologies (Berndt and Holzhütter, 2013; Iijima-Ando and Iijima, 2010; Kay et al., 2018; Lee and Lu, 2014; López–Doménech et al., 2018). In the present study, we used transgenic AD fly models expressing AD-related genes such as *Tau*, *Aβ_42_* and *Appl*, and demonstrated their possible genetic interaction with the *Drosophila Miro* gene. The genetic interaction study gives a new insight into understanding the complex mechanisms of AD as well as possible interactors of AD genes.

### Preliminary mechanisms involved in *Tau*, *Aβ_42_* and *Appl* induced toxicity in *Drosophila* models of AD

As shown above, the *Drosophila* models of AD mimic various AD-related pathologies. The rough eye phenotype (Fig. 1A–E,A′–E′,a–e) and defective phototaxis activity (Fig. 1F) shown by AD model flies were reported by previous studies showing that expression of AD associated genes results in accumulation of Aβ_42_ plaques and neurofibrillary tangles which cause excessive cell death in fly retina and results in the degeneration of photoreceptor cells (Ferreiro et al., 2018; Higham et al., 2019; Iijima-Ando and Iijima, 2010; Pak, 2010). Further, we observed defective climbing activity, reduced median lifespan and decreased body weight in AD model flies (Fig. 1G–I). This might be due to the excessive cell death occurring in the AD model flies as seen in AO stained third instar larval eye imaginal discs (Fig. 4D,G,J). Further, the phenotypic manifestation seen in AD model flies was correlated with increased mitochondrial/cellular ROS (Fig. 5I–L,Q–T,a) and an increased expression of anti-oxidant enzymes (*Mn-SOD* and *CAT*) in AD as a result of the compensatory mechanism against the increased ROS level (Fig. 5c) (Flynn and Melov, 2013; Niedzielska et al., 2016). Several studies have also suggested that increased ROS levels in AD is associated with mitochondrial damage, altered mitochondrial dynamics, and reduced ATP level (Castellani et al., 2002; Huang et al., 2016; Manoharan et al., 2016; Panchal and Tiwari, 2018). Thus, we examined the mitochondrial dynamics and observed fragmented mitochondria (reduced mitochondrial average length) (Fig. 6C,E,G) along with decreased expression of mitochondrial fusion related gene *Mitofusin* (*Mfn*) (Fig. 6H) in the AD model flies that ultimately results in reduced ATP level in AD model flies (Fig. 7A). This is in accordance with studies suggesting that expression of AD- related genes in *Drosophila* induced excessive cell death, higher oxidative stress and ATP deficiency that results in altered climbing activity, reduced body weight and reduced median lifespan (Keating, 2008; Lee et al., 2016; Ray et al., 2017; Winklhofer and Haass, 2010).

Further, the increased apoptosis seen in the larval brain of AD model flies (Fig. 8A–G,A′–G′) might be due to the higher oxidative stress and increased neurodegeneration. This was supported by the previous study by Wu et al. (2017) showing that increased apoptosis induced neurodegeneration in fly brain. Together these results suggest that expression of AD-associated genes (*Tau*, *Aβ_42_* and *Appl*) induced AD-related pathologies such as rough eye phenotype, defective behaviors (phototaxis and climbing), increased cell death, oxidative stress and neurodegeneration in *Drosophila*.

### Possible mechanisms underlying *Tau*, *Aβ_42_* and *Appl* induced toxicity modulated by overexpression of *Miro* in AD model flies

There was improvement seen in the AD-related pathologies such as rough eye phenotype and phototaxis activity in *Miro* overexpressing genetic background (Fig. 2A–D,A′–D′,J) and increased pathology and behavioral deficits in *Miro* knockdown flies (Fig. 2E–H,E′–H′,J). These results are supported by a previous study by Iijima-Ando et al. (2012) that demonstrated that the knockdown of *Miro* increases Tau mediated toxicity via increasing the accumulation of hyperphosphorylated Tau via PAR1 kinase activation. Thus, toxicity induced by the abnormal accumulation of Tau in AD model flies might lead to an increase in rough eye phenotype along with defective phototaxis activity in *Miro* knockdown flies. These improvements might be due to the overexpression of *Miro* that reduced cell death in eye imaginal discs (Fig. 5E,H,J). Thus, overexpression of *Miro* might help in the reduction of neurodegeneration of photoreceptor neurons, improvement in the rough eye phenotype and phototactic behavior (Cutler et al., 2015; Gistelinck et al., 2012; Prüßing et al., 2013; Wang and Montell, 2007).

The overexpression of *Miro* also improved the climbing activity (Fig. 2K), increased the body weight (Fig. 2L) and median lifespan (Fig. 3) associated with AD model flies. This might be due to the cumulative effect of reduced Aβ_42_ induced toxicity, and reduction in cell death and oxidative stress due to the overexpression of *Miro* in AD flies (Gorman, 2008; Niikura et al., 2006).

Further, decreased mitochondrial (Fig. 5M–P,U–X) and cellular (Fig. 5b) ROS level examined in the *Miro* overexpressing AD model flies might be associated with a regulatory role of *Miro* in the maintenance of mitochondrial structural integrity by reducing the toxicity associated with expression of AD-related genes in *Drosophila*. This result was supported by the previous study showing that inhibition of abnormal mitochondrial fission and mitochondrial dysfunction could significantly reduce ROS level (Tönnies and Trushina, 2017; Wang et al., 2014). It is suggested that *Miro* might help in the maintenance of mitochondrial dynamics and their proper function. Further, we have checked the effect of *Miro* overexpression on mitochondrial dynamics in AD model flies. We observed that overexpression of *Miro* increased mitochondrial average length (Fig. 6D,F) that was associated with increased expression of the mitochondrial fusion gene, *Mitofusin* (*Mfn*) in AD model flies (Fig. 6H). The increased mitochondrial length in *Miro* overexpressing flies was correlated with increased mitochondrial fusion related protein ‘Mitofusin’ (Lin and Sheng, 2015; Panchal and Tiwari, 2018). Thus, it was inferred that overexpression of *Miro* increases the mitochondrial length and improves the mitochondrial dynamics with decreased ROS level in AD model flies.

Moreover, the increased ATP level seen in *Miro* overexpressing AD model flies (Fig. 7A) is supported by the previous studies showing that mitochondria fusion is involved in increasing ATP production (Mitra et al., 2012; Song and Hwang, 2019). Thus, the increased ATP level along with reduced ROS subsequently improves the motor function, climbing activity, body weight and lifespan of AD model flies.

Moreover, the reduced number of apoptotic cells seen in third instar larval brain of *Miro* overexpressing AD model flies (Fig. 8A–F,A′–F′) might be due to the decreased oxidative stress and increased ATP level resulting from *Miro* overexpression in AD model flies (Fernández-Moriano et al., 2015; Santos et al., 2010).

Furthermore, the histological analysis of adult fly brains suggests that neurodegeneration in *Miro* overexpressing AD model flies was significantly decreased (Fig. 8A″–F^-^). This result was supported by the previous study demonstrating that reduced cell death decreases the neurodegeneration in the fly brain (Cai and Tammineni, 2017; Pathak et al., 2013).

### Conclusion

We demonstrated that overexpression of *Miro* modulates the AD-related pathologies in fly models of AD by decreasing the rough eye phenotype, improving the behavior defects such as phototaxis and climbing activity along with reducing apoptosis, increasing the ATP level and decreasing neurodegeneration in the AD model flies. Based on these observations, we conclude that the mitochondrial axonal transport gene *Miro* genetically interacts with AD-associated genes (*Tau*, *Aβ_42_*and* Appl*) in *Drosophila* and is a potential target for therapeutic intervention for neurodegenerative diseases.

## MATERIALS AND METHODS

### Fly stocks and genetics

*OregonR^+^* is a wild-type strain of *D.* *melanogaster*. GAL4 fly stocks: Pan-retinal *GMR-GAL4* [Chromosome (ChrII)] drives the expression of the genes in all cells posterior to the morphogenetic furrow (MF) in the developing eye and later on it becomes active throughout most of the pupal eye (Ellis et al., 1993; Freeman, 1996), Pan-neuronal *elav-Gal4^C155^* (ChrX) [Bloomington number (BL# 458)] drives the expression of genes in the neurons of the fly brain under elav control. Both of these flies (*GMR-GAL4* and *elav-Gal4^C155^*) were used as an experimental control.

In this study, AD genes were crossed with e*lav^C155^-GAL4* to express AD causing genes in the neurons, which induces degenerative phenotypes, such as pathological morphologies and behavioral changes. AD-associated genes were also expressed in the fly eye using the *GMR-GAL4* driver, which induced retinal degeneration that is indicated as rough eye phenotype.

*Miro* overexpressing/knockdown transgenic fly stocks: *UAS-Miro* (ChrII) (BL# 51646) overexpresses the *Miro* gene (Russo et al., 2009) and UAS*-Miro^RNAi^* (ChrIII) (BL# 43973) is an RNA interference (RNAi) line of *Miro* gene induced Miro knockdown line. Transgenic fly stocks overexpressing or knocking down AD-related genes: *UAS-Tau_WT_* (ChrII) (BL# 51362) expresses wild-type *Tau* under the control of UAS, *UAS-Aβ_42_(Human)/CyO* (ChrII), expressed human *Aβ_42_* gene under the control of UAS,*UAS-Appl^RNAi^* (ChrIII) (BL# 28043) is an RNAi line of *Appl* gene, w*;*GMR-GAL4-UAS-TAU_WT_/CyO;+/+* is a recombined fly stock of *GMR-GAL4* with *UAS-TAU_WT_/CyO*, w*;*GMR-GAL4-UAS-Aβ_42_(Human)/CyO;+/+* is a recombined fly stock of *GMR-GAL4* with *UAS-Aβ_42_ (Human)/CyO*.

*UAS-Aβ_42_E693G* (ChrIII) (BL# 33774) expressed the human Abeta42 fragment of APP carrying the familial Alzheimer's ‘Arctic’ mutation (E693G - amino acid numbering based on APP sequence) under the control of UAS. *UAS-APP.C99-UAS-MAPT* (ChrIII) (BL# 33803) expresses the C99 fragment of APP with the human APP signal peptide and human MAPT (tau) under the control of UAS.

We used the *UAS-Aβ_42_E693G* and *UAS-APP.C99-UAS-MAPT* fly strain for climbing and survival assays, body weight, ROS and ATP level measurement, mitochondrial dynamics, cell death and neurodegeneration analysis because the other AD transgenic flies such as *UAS-Tau_WT_*, *UAS-Aβ_42_(Human)/CyO* and *UAS-Miro* flies are located on the second chromosome. Therefore, it was not possible to cross each AD-related gene and *UAS-Miro* with e*lav^C155^-GAL4*.

GFP tagged mitochondria fly stock: *UAS-Mito-GFP/CyO* (ChrII) is a transgenic fly line expressing a GFP tagged N-terminal mitochondrial localization signal (Wang and Schwarz, 2009). w*;*GMR-GAL4-UAS-Mito-GFP/CyO;+/+* is a recombined fly stock of *UAS-Mito-GFP* with *GMR-GAL4*.

All flies were maintained at 22±1°C in a BOD incubator on standard *Drosophila* food media containing agar–cornmeal–sugar–yeast, nepagin (anti-fungal agent) and propionic acid (anti-fungal agent).

### Light microscopic imaging

For light microscopic imaging of the eyes of flies, 10-day-old adult flies from control and AD model flies were taken. Flies were anesthetized and eye images were captured at 51.2X magnification using a Carl Zeiss Stemi™ DV4 stereo binocular microscope with TSView7 software (version 7.1.3.7), which is expressed in micrometers. A total of 50 flies from each genotype were used for light microscopic imaging.

### Scanning electron microscopy (SEM)

SEM was performed to examine the detailed external morphology of *Drosophila* eyes as described by Iyer et al. (2016) with slight modifications. Briefly, 10-day-old flies of desired genotypes were decapitated and fixed in 2.5% glutaraldehyde (cat# G5882, Sigma-Aldrich, USA) prepared in 0.2 M sodium cacodylate (cat# C0250, Sigma-Aldrich, USA) buffered overnight at 4°C followed by three washes with 0.1 M PBS, 15 min each at room temperature (RT). Samples were immediately dehydrated in a graded series of ethanol (50%, 70%, 80% and 100%) and freeze-dried using lyophilizer (FreeZone, Labconco, USA). The dried samples were mounted on carbon taped SEM stubs and sputter coated with platinum for 90 s. Images were taken using a SEM (Jeol-JSM-7600F, Japan). A total 50 flies from each genotype were used for SEM study.

### Phototaxis assay

The Phototaxis assays were performed as described by Panchal and Tiwari (2017). For this, 10-day-old flies of desired genotypes were added to a Y-maze tube (Y-maze tube possesses one light arm and one dark arm) and allowed to acclimatize for 2 min in the tube. Flies were tapped gently to the bottom of the tube and allowed to move through the Y-maze tube for 20 s and the number of flies moving along the light and dark paths were counted. 20 flies of each genotype were placed in the Y-maze tube at a time and the experiment was repeated five times. A total of 100 flies from each genotype were used for phototaxis assay. The assay was performed under standard lighting conditions (∼500 Lux) and the phototaxis activity was presented as a light preference index= (number of flies that travelled along the light path - number of flies that travelled along the dark path/ total number of flies).

### Climbing assay

The climbing assay was performed as mentioned in Panchal and Tiwari (2017). For this, 10-, 20- and 30-day-old flies were placed in a vertical glass tube (30 cm long×1.5 cm wide) and allowed to acclimatize for 2 min. Flies were tapped gently to the bottom of the vial and the number of the flies crossing 8 cm 10 s^−1^ was counted. 20 flies of each genotype were placed in the glass tube at a time and the experiment was repeated five times. A total of 100 flies from each genotype were used. The assay was performed under standard lighting conditions. The climbing assay was expressed as % climbing 8 cm 10 s^−1^.

### Survival assay

The survival assay was performed as mentioned in Kumar et al. (2017). Briefly, the survival of adult flies was measured from the day of eclosion. Each vial of flies was transferred to fresh medium on every alternate day and the number of dead flies was counted until all flies were dead. A total of 100 flies were taken (20 flies/vial) for all genotypes. The median lifespan was calculated using the Kaplan–Meier method (Kaplan and Meier, 1958) and displayed as survival curves by using GraphPad Prism 5.0 software. The significant difference in the median lifespan between genotypes was assessed using Mantel-Cox (Mantel, 1966) log-rank test. The statistical data analysis was performed using GraphPad Prism 5.0 software.

### Body weight analysis

The body weight analysis was performed as mentioned in Panchal and Tiwari (2017) with little modification. For body weight analysis, 10-, 20- and 30-day-old flies were used for each genotype. The body weight of flies was measured by weighing 20 flies at a time using a weighing balance (Sartorius, Germany). The experiment was repeated five times. A total of 100 flies were taken for each genotype. Body weight of flies were measured in milligrams (mg).

### Quantitative real time PCR (RT-qPCR)

RT-qPCR was performed as described by Hwang et al. (2019) with slight modification. Briefly, mRNA from 30-day-old flies' heads were isolated using TRIzol reagent (cat. #15596026, Invitrogen, USA). cDNA was synthesized by using Verso cDNA Synthesis Kit (cat. #AB-1453/B, Thermo Fisher Scientific, USA) according to the manufacturer's protocol. cDNAs were amplified using the desired gene specific primers. A total of 20 µl of reaction mixer was prepared by adding cDNA, primers and PowerUp™ SYBR™ Green Master Mix (cat. #A25742, Applied Biosystem, Thermo Fisher Scientific, USA). Step one plus system (Applied BioSystems, USA) was used for RT-qPCR. Relative quantification was performed using the ′delta-delta Ct′ method to normalize with the *RP49* endogenous gene. Data are presented as Mean±s.d. (in the case of the *Miro* gene) and Mean±s.e.m. Relative levels of mRNA were analyzed by one-way ANOVA and analysis with Tukey's test was performed using GraphPad Prism 5.0 software.

The following primers were used: *Miro*(F): 5′-GGACGATGACGACACTTTGGA-3′, (R): 5′ CCAGGGAGGGATTGCACTT-3′; *Mitofusin (Mfn)* (F): 5′-TCTCGCAGAGTGCTGTGAAAA-3′, (R) 5′-CATGTCACCCGAAACACTCTTG-3′; *Mn-SOD*(F): 5′-CCAGACCTACGTCAACAATC-3′, (R) 5′-GATGGCCTTCTTCAGATCAT-3′; *CAT*(F):ACCAGGGCATCAAGAATCTG-3′, (R) 5′-AACTTCTTGGCCTGCTCGTA-3′. ATP synthase beta (F): 5′-TCCGCTTTGTTGGGTCGTA-3′, (R) 5′-CCATGTCGGTAGCCAAGGTT-3′.

### AO staining

AO staining was performed to examine the apoptotic cells as described by Kumar and Tiwari (2018) with slight modification. Briefly, third instar larval eye imaginal discs and third instar larval brain brains were dissected out in 1×phosphate-buffered saline (PBS) and incubated in 1 μg ml^−1^ AO solution (cat. #877529, Invitrogen, USA) prepared in 1X PBS for 2 min. After a brief wash in 1X PBS, the tissue was mounted in 1X PBS and immediately observed under the laser scanning confocal microscope (TCS SP5II, Leica Microsystems, Wetzlar, Germany). A total of 20 third instar larval eye imaginal discs and larval brains were taken for each genotype. Quantification of AO positive cells was measured by using ImageJ 5.0 software (NIH, USA).

### Measurement of mitochondrial and cellular ROS

Mitochondria superoxide (ROS) was measured using the ROS-sensitive MitoSOX™ Red staining (cat. #M36008, Invitrogen, USA) as prescribed in Liu et al. (2013). MitoSOX is a DHE derivative that possesses a cationic triphenylphosphonium group (TPP+), which helps in the transport to the mitochondrial matrix (Fuentes-Retamal et al., 2020; Roelofs et al., 2015). In the presence of ROS, MitoSOX gets oxidized and emits red fluorescence which was used to measure the ROS production (Forkink et al., 2010; Sarmiento-Salinas et al., 2019). For this, third instar larval brains from desired genotypes were dissected in cold Hanks' Balanced Salt Solution (HBSS) and incubated in 5 µM MitoSOX Red and 1 µM MitoTracker Green FM (cat. #M7514, Invitrogen, USA) for 20 min at 37°C. After removing MitoSOX Red and MitoTracker Green solutions, brains were washed with HBSS twice and mounted in 1 X PBS. The images were captured using laser scanning confocal microscope (TCS SP5II, Leica Microsystems, Wetzlar, Germany). The detection of the colocalization of MitoSOX Red and MitoTracker Green was done by observing the yellow fluorescence in the overlay images. A total of 20 larval brains were examined for each genotype. All brains used for immunofluorescence were examined using a laser scanning confocal microscope (TCS SP5II, Leica Microsystems, Wetzlar, Germany) and MitoSOX Red fluorescence intensity was measured by using ImageJ 5.0 software, NIH, USA. Cellular (cytosolic) ROS was measured using redox sensitive fluorophore DCF-DA dye (cat. #D399, Thermo Fisher Scientific, USA) as described in Westfall et al. (2018). Briefly, the fresh-pooled *Drosophila* homogenates were prepared from 30 heads of 30-day-old flies of desired genotype flies in Tris-EDTA-TritonX-100 buffer (pH 7.4) with a pellet pestle on ice. The homogenate was centrifuged at 12,000 rpm for 15 min at 4°C. The supernatant was collected for quantification of 2,7-dichlorofluorescein (DCF) fluorescence and 20 μl of fly homogenate with 170 μl of Locke's buffer and 10 μl of 1 mM DCF-DA solution were added in each well of a 96-well plate and incubated for 15 min at RT. The DCF-DA fluorescent signal was analyzed by 488_nm_/527_nm_ excitation/emission in a multimode microplate reader (SpectraMax^®^M2e, Molecular Devices, USA). The assay was performed in triplicates. Quantification was normalized to the amount of protein in each sample. The concentration of protein from the samples was also determined using the Bradford reagent (cat. #B6916, Sigma-Aldrich, USA).

### Mitochondrial average length measurement

The average length of mitochondria was measured using GFP tagged mitochondria expressing fly stock *GMR-GAL4-UAS-Mito-GFP/CyO*. This was done by dissecting third instar larval eye imaginal discs of desired genotype flies in 1X PBS and incubating them in 4% paraformaldehyde (PFA in 1XPBS) for 30 min at RT followed by washing with 1X PBS three times for 5 min each. The eye discs were mounted in 1, 4-Diazabicyclo [2.2.2] octane (DABCO, Sigma-Aldrich, USA), an antifade mounting medium and observed under the laser scanning confocal microscope (TCS SP5II, Leica Microsystems, Wetzlar, Germany). A total of 20 third instar larval eye imaginal discs were taken for each genotype.

### ATP Quantification

ATP quantification was performed as mentioned in Tennessen et al. (2014). Briefly, the fresh pooled *Drosophila* homogenates were prepared from 30 heads of 30-day-old flies of desired genotypes in 100 µl of homogenization buffer [6 M guanidine HCL, 100 mM Tris (pH 7.8), 4 mM EDTA] with a pellet pestle on ice. Samples were centrifuged at 12,000 rpm for 15 min to remove the debris, and the supernatant was diluted (1:750) in dilution buffer [25 mM Tris (pH 7.8), 100 µM EDTA]. The diluted homogenate was centrifuged at 12,000 rpm and 10 µl of the supernatant was transferred to individual wells of a white, opaque 96 well plate (cat. #3362, Corning, USA). A series of ATP standards were prepared by diluting the 5 mM ATP stock solution provided with an ATP bioluminescence assay kit (cat. #A22066, Invitrogen, USA) with ddH_2_O (0, 0.01, 0.05, 0.1, 0.5, 1 µM). 10 µl of each ATP standard solution used for the standard curve. The assay was started by adding 100 µl of the luciferase reaction mix and measuring the luminescence at 560 nm with a plate reader (Centro LB 960, Berthold Technologies, Germany). The assay was performed in triplicates. The concentration of protein from the samples was also determined using the Bradford reagent (cat. #B6916, Sigma-Aldrich, USA) and the ATP level was normalized to the protein content.

### Histological analysis

Histology analysis was performed as described in Iijima-Ando et al. (2012) with little modification. Briefly, to analyze the neurodegeneration, heads of 30-day-old flies were fixed in Bouin's fixative for 48 h at RT and incubated in 50 mM Tris/150 mM NaCl for 24 h. The tissues were processed in 10% formalin, ascending concentration of IPA (70%, 80%, 90% and 100%), xylene and infiltrated with paraffin wax at 65°C. Then tissues were subsequently embedded in paraffin wax. Serial sections (4 µm thickness) through the entire heads were taken on poly-L-lysine (cat. #P8920, Sigma-Aldrich, USA) coated glass slides using a microtome (HistoCore AUTOCUT, Leica, Germany). The tissues were stained with Hematoxylin (nucleus) and Eosin (cytoplasm) and examined under laser scanning confocal microscope (TCS SP5II, Leica Microsystems, Wetzlar, Germany). A total of ten adult flies brains were taken for each genotype. The numbers of vacuoles were counted to see the extent of neurodegeneration using ImageJ 5.0 software (NIH, USA).

### Immunostaining of larval brains

The immunostaining of larval brain was performed by selecting third instar larval brains from desired genotypes and dissecting them in 1X PBS followed by fixation in 4% PFA for 30 min at RT. The brains were washed in 1% PBST (1X PBS, 1% Triton X-100) three times, 15 min each and blocked in blocking solution [4% bovine serum albumin (BSA) solution in 1X PBS] for 2 h at RT followed by incubation in primary antibody rabbit anti-cleaved caspase-3 (Asp175) (5A1E) (1:150, cat. #9664, Cell Signaling Technology, USA) in blocking solution for overnight at 4°C. The brains were washed with 0.1% PBST (1X PBS, 1% Triton X-100) three times, 15 min each and blocked in blocking solution for 1 h at RT followed by secondary antibody incubation, anti-rabbit, IgG conjugated with Cy-3 (1:100, cat. #C2306, Sigma-Aldrich, USA), for 2 h at RT and washed with 0.1% PBST three times, 15 min each followed by mounting in DABCO (Sigma-Aldrich, USA) an antifade mounting medium. The samples were examined under the laser scanning confocal microscope (TCS SP5II, Leica Microsystems, Wetzlar, Germany). A total of 20 third instar larval brains were taken for each genotype. The fluorescence intensity of cleaved caspase-3 was measured using ImageJ 5.0 software (NIH, USA).

### Statistical analysis

All data are represented as Mean±s.e.m. except Fig. 4K RT-qPCR data, which are shown as Mean±s.d. For survival assays, the Kaplan–Meier method and Mantel-Cox tests were performed using GraphPad Prism 5.0 Software (San Diego, CA, USA). The biological replicates are shown as *n*. Significance between genotypes for all experiments was analyzed by one-way ANOVA analysis with Tukey's test using GraphPad Prism 5.0 for all data except survival assays. All images were assembled using Adobe Photoshop 7.0^®^.The histograms for all data were prepared by using GraphPad Prism 5.0 software and significance indicates as: ns, non-significant; *P<0.05, **P<0.01, ***P<0.0001.
